# 1,2,4-Trioxolane and 1,2,4,5-Tetraoxane Endoperoxides against Old-World *Leishmania* Parasites: In Vitro Activity and Mode of Action

**DOI:** 10.3390/ph15040446

**Published:** 2022-04-03

**Authors:** Andreia Mendes, Ana Armada, Lília I. L. Cabral, Patrícia S. M. Amado, Lenea Campino, Maria L. S. Cristiano, Sofia Cortes

**Affiliations:** 1 Instituto de Higiene e Medicina Tropical (IHMT), Universidade Nova de Lisboa (UNL), Rua da Junqueira, 100, 1349-008 Lisboa, Portugal; andricia.mormendes@gmail.com (A.M.); aarmada@ihmt.unl.pt (A.A.); lcampino@ihmt.unl.pt (L.C.); 2 Global Health Tropical Medicine (GHTM), Instituto de Higiene e Medicina Tropical (IHMT), Universidade Nova de Lisboa (UNL), Rua da Junqueira, 100, 1349-008 Lisboa, Portugal; 3 Centro de Ciências do Mar (CCMAR), Universidade do Algarve, Campus de Gambelas, 8005-139 Faro, Portugal; liliacabral80@gmail.com (L.I.L.C.); patricia.s.amado@gmail.com (P.S.M.A.); 4 Departamento de Química e Farmácia, Faculdade de Ciências e Tecnologia, Universidade do Algarve, Campus de Gambelas, 8005-139 Faro, Portugal

**Keywords:** *Leishmania infantum*, *Leishmania donovani*, leishmaniasis, 1,2,4-trioxolanes, 1,2,4,5-tetraoxanes, selectivity, mode of action, reactive oxygen species

## Abstract

Leishmaniasis remains one of the ten Neglected Tropical Diseases with significant morbidity and mortality in humans. Current treatment of visceral leishmaniasis is difficult due to a lack of effective, non-toxic, and non-extensive medications. This study aimed to evaluate the selectivity of 12 synthetic endoperoxides (1,2,4-trioxolanes; 1,2,4,5-tetraoxanes) and uncover their biochemical effects on *Leishmania* parasites responsible for visceral leishmaniasis. The compounds were screened for in vitro activity against *L. infantum* and *L. donovani* and for cytotoxicity in two monocytic cell lines (J774A.1 and THP-1) using the methyl thiazol tetrazolium assay. Reactive oxygen species formation, apoptosis, and mitochondrial impairment were measured by flow cytometry. The compounds exhibited fair to moderate anti-proliferative activity against promastigotes of the 2 *Leishmania* species, with IC_50_ values ranging from 13.0 ± 1.7 µM to 793.0 ± 37.2 µM. Tetraoxanes LC132 and LC138 demonstrated good leishmanicidal activity on *L. infantum* amastigotes (IC_50_ 13.2 ± 5.2 and 23.9 ± 2.7 µM) with low cytotoxicity in mammalian cells (SIs 22.1 and 118.6), indicating selectivity towards the parasite. Furthermore, LC138 was able to induce late apoptosis and dose-dependent oxidative stress without affecting mithocondria. Compounds LC132 and LC138 can be further explored as potential antileishmanial chemotypes.

## 1. Introduction

Leishmaniases are neglected vector-borne infectious diseases caused by protozoan parasites of the genus *Leishmania* that create impacting illness burden in 98 countries. The WHO estimates 12 million human infections worldwide, with over 1 million new cases annually, leading to different forms of the disease, namely cutaneous (CL), mucocutaneous (MCL), or visceral leishmaniasis (VL). The latter occurs in up to 90,000 cases and is fatal if untreated, with an estimated 65,000 deaths per year [1]. *L. infantum* and *L. donovani* are the main aetiological agents of VL in the Old World, with an anthroponotic and zoonotic transmission, respectively. The major aetiological agents of CL are *L. major* and *L. tropica,* characterised by zoonotic and anthroponotic transmission, respectively. Although less frequently, *L. infantum* can also cause CL. *Leishmania* sp. parasites present a digenic life cycle that alternates between a mammalian host and a phlebotomine insect vector, usually responsible for disease transmission.

Given the absence of vaccines to prevent leishmaniases in humans and their low effectiveness for canine leishmaniases, disease control relies mostly on chemotherapy. Unfortunately, limited investment from the funding agencies and pharmaceutical companies has been directed to the development of new molecules and to support clinical trials towards novel therapies for leishmaniases [2]. Currently available drugs for treating human leishmaniases, in use for several decades, are pentavalent antimonials, amphotericin B (AmB), miltefosine, and pentamidine. Despite their effectiveness, these drugs are often associated with high toxicity, adverse side effects, high cost, and long-term periods of parenteral administration [3]. In addition, evidence of parasite resistance presents a major concern [4], and thus the search for safe, non-toxic, and cost-effective antileishmanial drugs is of utmost urgency. In the last decades, research in the field unravelled relevant drug targets and yielded promising leads for developing new drugs. However, none has yet materialized into newly available drugs or drug regimens. Consequently, the search for novel antileishmanial candidates remains a priority.

Artemisinin and derivatives (ART’s) and other endoperoxides from synthetic origin (trioxanes, trioxolanes, and tetraoxanes) have demonstrated antiparasitic activity in different protozoan parasites, namely against *Plasmodium* sp. [5], *Toxoplasma gondii*, *Trypanosoma* sp. and *Schistosoma mansoni* [6,7]. Early studies concerning the effect of endoperoxides on *Leishmania* sp. revealed the antiparasitic activity of a small selection of 1,2,4-trioxolanes. The compounds reduced *L. infantum* viability in host-stage (amastigotes) and vector-stage (promastigotes) forms of the parasite and presented attractive safety profiles, also highlighting the role of the endoperoxide bridge in activity [8]. Other studies have also demonstrated the activity of endoperoxides against *L. donovani* and *L. amazonensis*, presenting evidence of parasites metabolism inhibition and limited cytotoxicity for host cells [5,9,10]. A significant advantage of synthetic endoperoxide-containing compounds such as 1,2,4-trioxolanes and 1,2,4,5-tetraoxanes is their accessibility, which enables the preparation of chemically diversified libraries of analogues and a more precise selection of a possible lead compound [11].

The modes of bioactivation and action of ART’s against *Plasmodium* sp. were extensively investigated and the results have highlighted the central role of the peroxidic core. Evidence indicates that the peroxide (O–O) bond is reductively activated through the interaction with heme [Fe(II)] released during parasite haemoglobin digestion, leading to cleavage of the endoperoxide ring and consequent formation of reactive species that react with relevant parasite targets, e.g., proteins or lipids, through alkylation or peroxidation, promoting parasite death [5]. Contrarywise, the mechanisms of action of endoperoxides in *Leishmania* parasites were scarcely investigated. Earlier studies indicated that artemisinins induce a redox imbalance through formation of reactive oxygen species (ROS), pointing to a caspase-independent apoptotic mode of cell death in *Leishmania* promastigotes [9,12].

In the present work we disclose the anti-leishmanial potential of a chemically diverse library of synthetic endoperoxides comprising six 1,2,4-trioxolanes and six 1,2,4,5-tetraoxanes against different *Leishmania* spp., also providing further insights into the mode of action of these compounds.

## 2. Results

### 2.1. Cytotoxicity and In Vitro Activity of Peroxidic Compounds against Leishmania sp.

To evaluate the effect of 1,2,4-trioxolanes and 1,2,4,5-tetraoxanes on host cells, two mammalian macrophage cell lines were used, murine J774A.1 and human THP-1. Compounds presented variable cytotoxicity after 48 h, detected by methyl thiazol tetrazolium (MTT) colorimetric assay profiles in these cell lines, with half-maximal cytotoxic concentrations (CC_50_) ranging from 12.0 ± 0.5 µM to 1465 ± 13 µM for J774A.1 and from 78.2 ± 14.1 µM to 3569 ± 22 µM for THP-1 (Table 1). In general, peroxides proved more toxic for murine cells than for human cells. AmB and miltefosine, used as control drugs, presented CC_50_ values of 71.9 ± 5.4 µM and 94.4 ± 17.3 µM, respectively.

Promastigotes of *L. infantum* IMT151 and *L. donovani* BD14 were treated with the peroxidic compounds and the standard drugs. The compounds exhibited fair to moderate anti-proliferative activity against the two *Leishmania* species, measured by the MTT assay, with half-maximal inhibitory concentrations (IC_50_) ranging from 12.9 ± 1.7 µM to 491.6 ± 22.7 µM in *L. infantum*, and 14.3 ± 5.5 µM to 793.0 ± 37.2 µM in *L. donovani* (Table 1). Selectivity index (SI) is indicative of compounds’ safety, representing the ratio between the cytotoxicity and the parasite’s inhibitory concentrations (CC_50_/IC_50_). Table 1 shows that only LC132, LC137 and LC138 present SI values higher than 7, when considering the human cell line. When comparing with control drugs, SI values of c.a. 12 and 26 exhibited by compounds LC137 and LC138, for both viscerotropic *L. infantum* and *L. donovani* strains, were lower than those for AmB but considerably higher than those exhibited by miltefosine.

Compounds LC132, LC137 and LC138, which presented good selectivity on promastigote forms for both *Leishmania* species, compared to the other peroxidic compounds (Table 1), were selected for evaluation against the amastigote stage. This assay mimics the initial stage of infection, after transmission of *Leishmania* sp. from the sandfly to host. LC132 proved highly active against intracellular amastigotes on both species, with IC_50_ of 13.2 ± 5.2 µM in *L. infantum* (SIs 3.5 and 7.6) and 9.4 ± 0.1 µM in *L. donovani* (SIs 4.9 and 66.3) (Table 2). LC138 was more active and selective in *L. infantum* amastigotes than in *L. donovani*, with IC_50_ of 23.9 ± 2.7 µM and SI of 22.1 and 118.6 for both cell lines, with better selectivity than the control drug miltefosine.

### 2.2. Flow Cytometry Analysis

Promastigote forms of *L. infantum* were used as the cellular model for flow cytometry assays and treated with the control drug AmB and the endoperoxides LC132, LC137 and LC138, as these compounds were the ones considered for the intracellular amastigotes. H_2_O_2_ (2 mM) was used as positive control for apoptosis.

**Figure 1:**
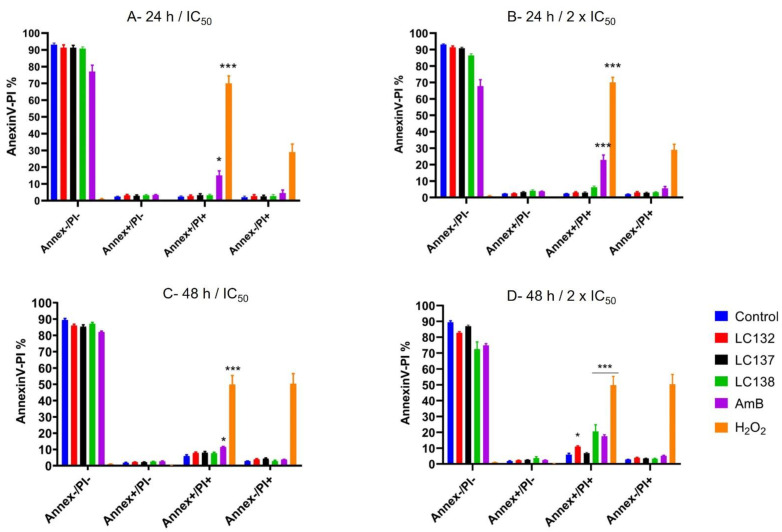
Flow cytometry analysis of phosphatidylserine externalization after 24 h (**A**,**B**) and 48 h (**C**,**D**) treatment of L. infantum promastigotes with IC_50_ LC132 (13.0 µM), LC137 (137.0 µM), LC138 (125.4 µM) or AmB (0.2 µM) and 2 × IC_50_ LC132 (25.9 µM), LC137 (274.0 µM), LC138 (250.8 µM or AmB (0.3 µM). Percentages correspond to Annexin V (Annex) and Propidium iodide (PI) cell populations: Annex−/PI−, live cells; Annex+/PI−, early apoptotic cells; Annex+/PI+, late apoptotic cells; Annex−/PI+, necrotic cells. 2 mM H_2_O_2_ was used as control for apoptosis. Data are represented by mean ± standard error of mean (SEM) of three independent experiments, with triplicates. Asterisks represent statistically significant differences of apoptotic populations related to Control (non-treated parasites) * *p* < 0.05; *** *p* < 0.0001).

Externalization of phosphatidylserine (PS) is considered as evidence of early apoptosis and can be detected through Annexin V (Annex) phospholipid-binding protein, which has high affinity for PS. To distinguish apoptotic from necrotic cell death, cells were counterstained with propidium iodide (PI). Promastigotes were treated with LC132, LC137, LC138 and AmB, at IC_50_ (13.0 µM, 137.0 µM, 125.4 µM, and 0.2 µM respectively) and 2 × IC_50_ (25.9 µM, 274.0 µM, 250.8 µM, and 0.3 µM, respectively), for 24 h and 48 h. At 24 h no significant differences were observed for the compounds, with most cells viable (Annex-/PI-), similar to control (Figure 1A,B). At 48 h, when parasites were subjected to IC_50_ no evidence was observed on early apoptosis (Annexin+/PI) (Figure 1C,D). However, an increase in late apoptosis was observed (Annexin+/PI+) with 2 × IC_50_, with significant differences in *L. infantum* promastigotes treated with LC138 (20.5 ± 4.2%) and AmB (17.5 ± 0.9%), compared to non-treated control (Figure 1D).

**Figure 2:**
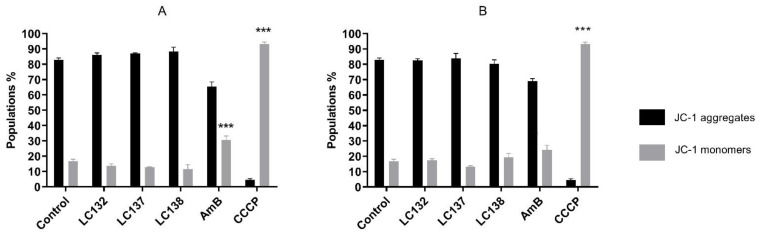
Mitochondrial membrane potential of L. infantum promastigotes incubated for 24 h with (**A**) IC_50_ (13.0 µM LC132, 137.0 µM LC137, 125.4 µM LC138 and 0.2 µM AmB) and (**B**) 2 × IC_50_ (25.9 µM LC132, 274.0 µM LC137, 250.8 µM LC138 and 0.3 µM AmB). Results are presented as percentage of cells with red fluorescence at ~590 nm (JC-1 aggregates—black bars—indicating dye accumulation in mitochondria) and green fluorescence at ~519 nm (JC-1 monomers—grey bars—indicating membrane depolarisation). Positive control for membrane depolarization corresponds to parasites treated with 50 μM carbonyl cyanide 3-chlorophenylhydrazone (CCCP). Data are represented by mean ± SEM of three independent experiments, with triplicates. Asterisks represent statistically significant differences of treated related to non-treated parasites; *** *p* < 0.0001.

The cationic dye 5′,6,6′-tetrachloro-1,1′,3,3′-tetraethylbenzimidazolylcarbocyanine iodide (JC-1) was used to acess the compounds effect on parasites mitochondrial membrane potential, 24 h post-treatment (Figure 2). Parasites treated with IC_50_ and 2 × IC_50_ of the three compounds presented similar behaviour to non-treated parasites, showing high population percentage of JC-1 aggregates (>80%), with no evidence of mitochondrial depolarization, meaning that mitochondria seem not to be affected by these compounds. Only treatment with AmB showed some effect on mitochondrial membrane potential, with a decrease in JC-1 aggregates (64.9 and 69.1%, Figure 2A,B).

**Figure 3:**
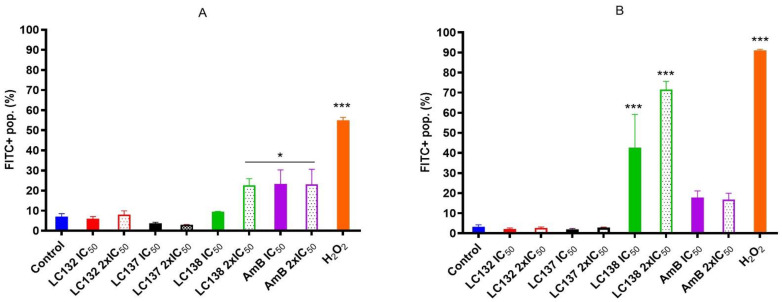
Effect of LC132, LC137, LC138 and AmB IC_50_ (13.0 µM, 137.0 µM, 125.4 µM and 0.2 µM, respectively) and 2 × IC_50_ (25.9 µM, 274.0 µM, 250.8 µM and 0.4 µM, respectively) on generation of ROS in L. infantum promastigotes, after (**A**) 5 h and (**B**) 24 h incubation. Parasites were labelled with CM-H_2_DCFDA. H_2_O_2_ (1 mM) was used as positive control. Data are represented by mean percentage of positive populations of FITC population ± SEM of three experiments, with triplicates. Asterisks represent statistically significant differences of treated, related to non-treated control * *p* < 0.01, *** *p* < 0.0001.

To evaluate how cells respond to oxidative stress in the first hours upon treatment, we used the ROS probe 5-(and-6)-chloromethyl-2′,7′-dichlorodihydrofluorescein diacetate, acetyl ester (CM-H_2_DCFDA) to monitor oxidation through increase in fluorescence. A slight increase in ROS levels (FITC+) was observed in an early phase of exposure with LC138 (2 × IC_50_, 250.8 µM), but after 24 h exposure a significant increase (*p* < 0.0001) was observed for this compound, with 42.6 ± 16.5% and 71.6 ± 4.0% of FITC+ population, respectively, in comparison with the control group, and much higher than AmB (Figure 3B). The other two compounds presented no alterations in ROS levels.

Concerning genomic DNA fragmentation, as result of programed cell death, no alterations were observed in parasites treated with the three compounds.

## 3. Discussion

Current therapeutic approaches for leishmaniases continue to present limitations, including drug resistance [2]. In attempting to repurpose antimalarial leads several endoperoxide compounds have been identified, from which emerged the well-established artemisinin based antimalarial drugs and selected synthetic endoperoxide candidates, including 1,2,4-trioxanes and 1,2,4,5-tetraoxanes. Preliminary research has shown that selected endoperoxides are attractive therapeutic candidates against various protozoal infections, including leishmaniases and other trypanosomatid diseases [5,6,10,13].

In the present study we synthesised a representative library of endoperoxides comprising bridged 1,2,4-trioxolanes and 1,2,4,5-tetraoxanes. The compounds were evaluated for toxicity in human monocytic (THP-1) and mice macrophage (J774A.1) cell lines, then for antileishmanial activity against *L. infantum* and *L. donovani* promastigotes, two old world causative agents of VL. The compounds exhibited variable cytotoxicity levels upon incubation with human monocytic (THP-1) and mice macrophage (J774A.1) cell lineages, ranging from medium to low CC_50_, in which the human-derived cell line was less affected by most of the tested compounds, suggesting that human-derived cell lines are an appropriate model for antileishmanial drug development. Differences between these two cell lines were observed previously, in which J774A.1 macrophages proved more vulnerable to oritavancin toxicity than human THP-1 cells. This distinction is most likely due to the higher accumulation of the drug by J774A.1 than by THP-1 human cells [14].

Our results showed that *L. infantum* and *L. donovani* promastigotes are susceptible to all peroxides scrutinized, with a dose-dependent inhibition, although with differences in IC_50_ for both species. Although *L. infantum* IMT151 is known to be a virulent strain [15], it proved to be susceptible to most of the studied peroxidic compounds. In fact, this strain already evidenced susceptibility to other peroxides of the same family [8]. *Leishmania* promastigotes are commonly used for preliminary in vitro screening due to the ease of culture growth in laboratory environments, the requirement of small quantities of extracts, fractions, or compounds, and the fact that these assays do not require sophisticated instruments [16]. Among the endoperoxides studied, the 1,2,4-trioxolane LC132 and the 1,2,4,5-tetraoxanes LC137 and LC138 demonstrated moderate selectivity (>7) despite their differences in the core ring substitution at position C4”, with a bulky 1-phenyl-1*H*-tetrazolyl moiety in LC132, compared to -H substitution in LC137 and an ethyl-ester group in LC138. We determined the overall lipophilicity of all compounds prepared using the partition coefficient (ClogP). From the results, these compounds show similar ClogP values [ClogP = 4.05 (LC132); 4.19 (LC137); and 3.91 (LC138)], the highest among the compounds synthesized (Table 3). One of the key features of these molecules relates to the role of the peroxidic bridge, which is believed to play a role in the mechanism of action, as indicated from prior studies on the effect of artemisinin and its semi-synthetic derivatives in *L. infantum* [8]. Further, the compounds selected were tested for their activity against intracellular amastigotes. These clinically relevant parasite forms, which replicate within macrophages, have been used as a reference model for in vitro screening of hit molecules. In general, LC138 presented SI > 10, indicating low cytotoxicity and good efficacy. Noteworthy, SI > 20 is considered hit activity criteria for *Leishmania* sp. amastigotes in macrophages, assuming that the observed pharmacological activity is not due to cytotoxicity [17]. Thus, although LC138 exhibits an interesting selectivity, some optimisations should be considered for this molecule. Miltefosine and AmB were used as reference drugs. Miltefosine appears to be highly selective on both *L. infantum* and *L. donovani* strains, while other in vitro studies have also shown that AmB is significantly more active against *Leishmania* spp. parasites than miltefosine [8,18]. The liposomal formulation of AmB is nowadays the first-line choice for the treatment of VL in several Mediterranean countries [19]. Miltefosine, the sole oral drug available, has been used as first-line drug in the Indian subcontinent, but after decades of use the rate of relapse with miltefosine in this area has risen significantly, due to increasing resistance [20].

Given the essential function that the single mitochondria plays in apoptosis, this organelle has been suggested as a potential drug target for many compounds [21]. Several cell death phenotypes have been described in trypanosomatids, with apoptotic markers identified, including loss of mitochondrial membrane potential, cytochrome c release, PS externalization, abnormal DNA condensation and fragmentation [22,23]. Externalization of PS by apoptotic cells is a typical predictor of early apoptosis and in *Leishmania* promastigotes endoperoxides like artemisinin cause phosphatidylserine externalization, as a result of membrane depolarization [9].

Although some authors questioned the presence and distribution of PS among the several morphological forms of *Leishmania* sp. [24], in our study LC138 presented mild evidence of PS externalization, which was more evident in a late stage of apoptosis (at 48 h), though with a generation of ROS at long exposure times (24 h). This evidence indicates that LC138 causes oxidative stress by activating ROS production by the mitochondrion or the endoplasmic reticulum, which is consistent with late mitochondrial injury, as previously reported [25]. Other authors observed that artemisinin’s toxicity against *L. donovani* promastigotes is thought to be mediated by a redox imbalance, as a result of the production of ROS caused by the cleavage of its endoperoxide bridge, the process ending in a caspase-independent apoptotic mode of cell death [9,26]. The response of the control drug amphotericin B was consistent with a typical apoptosis-like event. Anti-leishmanial drugs such as amphotericin B and miltefosine are known to induce apoptosis [27].

Mechanistic studies on the action of artemisinin in *L. tarantolae* suggested that, likewise with *Plasmodium* sp., it exerts its action by generating free radicals which change the cellular redox balance, causing parasites’ death. It was also shown that in *Plasmodium* sp. the conversion of artemisinin to free radicals is essentially caused by cellular heme and iron [28]. From our results, the peroxides selected do not seem to affect mitochondria, as there was no evidence of mitochondrial depolarization. On the other hand, artemisinin has been implicated in a lack of mitochondrial membrane potential in *L. donovani* promastigotes, ascribed to cleavage of the endoperoxide bridge and thiols depletion [9,12].

Nucleus of treated parasites were probably not affected, as analysis of DNA fragmentation of *L. infantum* upon treatment with the compounds herein studied didn’t show any alterations. Plasma membrane alterations with vesicules and vacuolization in cytoplasm were observed by TEM (data not shown), being a common apoptosis-necrosis process that occurs in an attempt to rescue the life of ATP-deprived cells from the mitochondria [29]. Since *L. infantum* treatment with LC132 or LC138 did not affect plasma membrane permeability, with slight PS externalization, nor the mitochondria, and did not produce DNA fragmentation, cell death mechanism could be other than apoptotic-like or necrotic events.

## 4. Materials and Methods

### 4.1. Synthesis

**Figure 4:**
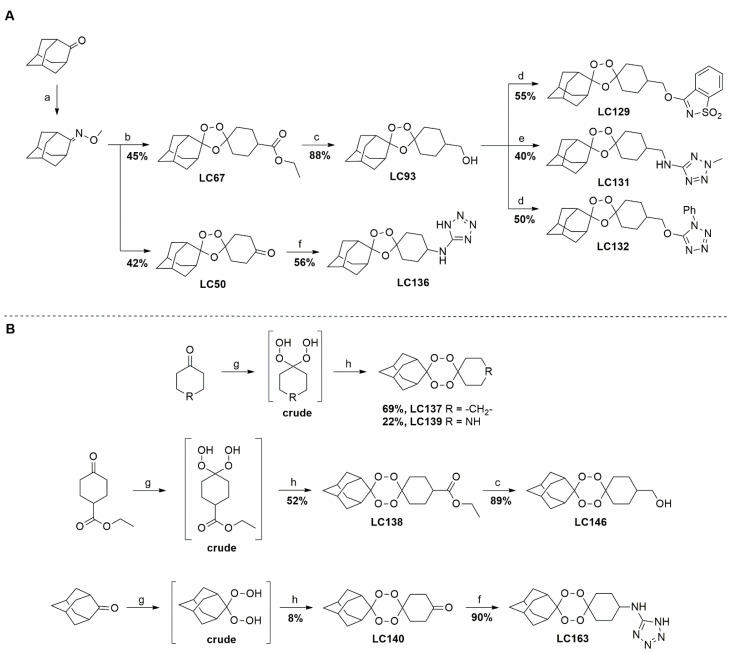
Synthesis and structural representation of the 1,2,4-trioxolanes and 1,2,4,5-tetraoxanes investigated in this work. (**A**) Representation of the synthetic routes to the 1,2,4-trioxolanes; reagents and conditions: (a) Pyridine, MeONH_2_, MeOH, r.t; (b) ketone, O_3_, CH_2_Cl_2_/Pentane, −78 °C; (c) 1. LiAlH_4_, anhydrous Et_2_O, 0 °C, 1 h; 2. H_2_O; (d) Chloride derivative, TEA, Toluene, 45 °C; (e) 1. Triethyamine, mesyl chloride, THF, 3 h, 2. Amine, 60 °C; (f) Amine, AcOH, DCE, NaBH(OAc)_3_, r.t.; (**B**) Representation of the synthetic routes to 1,2,4,5-tetraoxanes; reagents and conditions: (g) SSA, CH_3_CN, H_2_O_2_ 50% (*w/w*), 0 °C-rt; h) Ketone, SSA (2 eq), anhydrous CH_2_Cl_2_, rt, 2 h.

A representative library of 12 endoperoxides with potential antileishmanial action was designed and synthesized. Their structures, represented in Table 3, exhibit a peroxide pharmacophore (trioxolane or tetraoxane-based), flanked by adamantane and an appropriately functionalized cyclohexyl ring. The endoperoxides LC50, LC67, LC138, and LC140 were employed as intermediate compounds for expanding the diversity of analogues, by altering the cyclohexyl substitution in position C4’’. The endoperoxide-heterocycle conjugates (LC129, LC131, LC132, LC136, LC163) were synthesized through a convergent strategy, in which the endoperoxide and the corresponding heterocycle (tetrazole or saccharyl) building blocks were prepared independently and then coupled to generate the appropriate final targets (Figure 4). We developed or adapted the procedures for the synthesis of the 1,2,4-trioxolane- and 1,2,4,5-tetraoxane-derived conjugates [11,13,30,31]. Details of the synthesis and chemical characterization of the 12 peroxide-type compounds and their precursors are available as Supplementary Material.

### 4.2. Biological Studies

All compounds were dissolved in Dimethyl sulfoxide (DMSO, Sigma). Working solutions for the different biological studies were freshly prepared from the DMSO solutions and did not exceed 1% DMSO. AmB (Gibco) and Miltefosine (Sigma) were used as positive controls.

#### 4.2.1. Parasites and Cell Lines

Promastigote forms of strains of *L. infantum* (MHOM/PT/1988/IMT151) and *L. donovani* (MHOM/BD/2006/BD14) were cultured at 24 °C ± 1 °C in M199 medium (Sigma) supplemented with 20% foetal bovine serum (FBS, Sigma) and 1% penicillin-streptomycin (Sigma). *L. infantum* strain belongs to IHMT, Portugal cryobank collection and the *L. donovani* strain was kindly provided by Dr. Katrin Kuhls, Technical University of Applied Sciences Wildau, Germany. Promastigotes used for the experiments had 7 to 12 in vitro passages in culture after isolation from mice, in order to keep virulence, and were used when the stationary phase of growth was reached (day 5 to 6), corresponding to the highest promastigotes density.

Adherent BALB/c mouse macrophage cell line J774A.1 (ATCC^®^ TIB 67™) and suspension human acute monocytic leukemic cell line THP-1 (ATCC^®^ TIB-202™) were purchased from DSMZ, Germany were cultured and maintained in DMEM medium (Gibco) and RPMI 1640 (Sigma), respectively, supplemented with 10% FBS, 1% penicillin-streptomycin (Sigma) and L-glutamine 200 mM (Sigma), at 37 °C, 5% CO_2_.

#### 4.2.2. Cytotoxicity Assay

Cell lines J774A.1 and THP-1 were used to estimate CC_50_ of the peroxides. Cells were plated in 96-well flat bottom culture plates (VWR) at 5 × 10^5^ cells/mL. The monocytic cell line THP-1 was differentiated in RPMI medium with phorbol myristate acetate (PMA) (25 ng/mL; Sigma) for 24 h, followed by a washing step with RPMI medium. Six serial concentrations of the compounds (Table 3) were added in four replicates and the plates were incubated at 37 °C ± 1 °C, 5% CO_2_ for 48 h. An untreated control (cells in DMEM or RPMI medium plus 0.5% DMSO as vehicle) and blank (medium alone) were plated out in quadruplicate. After the incubation period, 20 µL/well of MTT [3-(4,5-dimethylthiazol-2-yl)-2,5-diphenyltetrazolium bromide] was added to a final concentration of 5 µg/mL in 1× phosphate-buffered saline (PBS, Merck), with an additional 4 h incubation at 36 °C, as previously described [32]. The formazan salts were resuspended in 2-propanol (Merck) and the cell viability was determined by measuring absorbance with a Synergy ™HTX Multi Mode Microplate Reader at 595 nm (Dynex Technologies). The CC_50_ values were calculated fitting the data as a non-linear regression with variable slope using a dose-response inhibitory model, in the GraphPad Prism V 8.0 program. Each compound was tested in three independent assays.

#### 4.2.3. Studies of In Vitro Activity against *Leishmania* spp.

##### Promastigotes Susceptibility Assay

For the determination of the antileishmanial activity and IC_50_, promastigotes of *L. infantum* and *L. donovani* were plated in 96-well flat bottom culture plates at a concentration of 5 × 10^6^ parasites/mL and incubated with six serial concentrations of each compound (Table 3), in four replicates, including the control drugs, amphotericin B, miltefosine and pentamidine. One untreated control (parasites with M199 medium plus 0.5% DMSO as vehicle solution) and one blank (M199 medium alone) were also plated out in quadruplicate. After 48 h incubation at 24 °C ± 1 °C, the inhibitory activity of the compounds was evaluated using the MTT, as previously described, with an additional 4 h incubation at 36 °C. Results were expressed in terms of percentage of parasite viability relative to nontreated control and IC_50_ was calculated fitting the data as a non-linear regression with variable slope using a dose-response inhibitory model, in the GraphPad Prism V 8.0 program. Each compound was tested in three independent assays.

SI was determined for each compound, expressed by the ratio between cytotoxicity in cell lines (CC_50_) and anti-*Leishmania* spp. activity (IC_50_) (SI = CC_50_/IC_50_).

##### Intracellular Amastigotes Assay

The intracellular assay using the macrophage J774A.1 cell line was performed with the compounds that presented best SI on the promastigote’s susceptibility assay. J774A.1 cells were adjusted to 2.5 × 10^5^ cells/mL in supplemented DMEM medium. 200 μL/well of the cell suspension was placed in 16 well Lab Tek culture glass slides (Nunc), and incubated at 37 °C ± 1 °C, 5% CO_2_, for 3 h, to enable the cells to adhere to the slides. After a washing step with DMEM to discard non-attached cells, the cells were infected with 2.5 × 10^6^ cells/mL promastigotes in a 10:1 parasite-host cell proportion [33] for 24 h. Compounds and control drugs were added in two replicates of six serial concentrations (Table 3). As negative control, macrophages were cultivated in medium and DMSO, as vehicle solution (0.5% *v/v*). Slides were incubated for 48 h at the same temperature. After a washing step with 1× PBS (Sigma), slides were fixed and stained, and the infection index was calculated according to [34] by counting the number of intracellular amastigotes per 100 macrophages, in treated and untreated cells. Two independent experiments were performed to determine the amastigotes IC_50_ of each compound, which was calculated by fitting the data as a non-linear regression with variable slope using a dose-response inhibitory model, in the GraphPad Prism V 8.0 program. SI was determined as described above.

#### 4.2.4. Flow Cytometry Analysis

The *L. infantum* strain (MHOM/PT/1988/IMT151) was chosen for the cytometry analysis and promastigotes at a concentration of 1 × 10^7^ parasites/mL were tested against the three compounds evaluated in the intracellular amastigotes assay (LC132, LC137 and LC138). Flow cytometry analysis were performed in a CytoFlex flow cytometer (Beckman Coulter), at an excitation wavelength of 488 nm (blue laser). Data acquisition were collected and registered using channels FL1 (flurescein isothiocyanate, FITC) with a BP filter (525 ± 40 nm) for Fluorescein and FL2 (IP) with a BP filter (570 ± 20 nm) for propidium iodide (PI) and 10,000 total events per sample were analysed using CytExpert v2.0.0.153 software. Samples were analysed in triplicates and two to three independent experiments were performed. Values are expressed as a percentage of cells for a given marker, relatively to the number of cells analysed ± standard error of mean (SEM).

##### Detection of Phosphatidylserine

To study the apoptosis inducing capacity of the most selective compounds, PS translocation from the inner to the outer leaflet of the plasma membrane was accessed, as it is a characteristic event of early apoptosis. To distinguish apoptotic cell death from necrotic cell death, cells were counterstained with PI, a non-permeable stain with affinity for nucleic acids, as it selectively enters necrotic cells. Parasites were stained with Annexin V and PI according to the manufacturer’s instructions (FITC Annexin V/PI Apoptosis Detection Kit I, BD Pharmingen). Briefly, *L. infantum* promastigotes were incubated with IC_50_ and 2 × IC_50_ of the tested compounds and amphotericin B, for 24 h and 48 h, at 24 °C ± 1 °C. H_2_O_2_ (2 mM) was used as positive control and nontreated parasites as negative control. In addition, amphotericin B was used as control drug. After a washing step with cold 1 × PBS (10 min, 800× *g*), cells were resuspended in 1 × Binding Buffer. 100 μL of cell suspension was incubated with 5 μL of Annexin V and 5 μL of PI for 15 min at RT in the dark. After adding 400 μL of 1 × Binding Buffer, samples were analysed in Cytoflex flow cytometer. Annexin V-FITC and PI were measured in FL1 and FL2 channels, respectively. The following controls were used to set up compensation and quadrants: unstained cells, cells stained with FITC Annexin V (no PI), cells stained with PI (no FITC Annexin V).

Parasites populations were characterized as follows: (1) viable: Annexin V-/Pi-; (2) early stages of apoptosis: Annexin V+/Pi−; (3) late stages of apoptosis: Annexin V+/Pi+ and (4) other type of cell death: Annexin V-/Pi+.

##### Analysis of the Mitochondrial Membrane Potential

Mitochondrial damage was evaluated through the analysis of the mitochondrial membrane potential with MitoProbe™ JC-1 Assay Kit for Flow Cytometry (Molecular Probes, Invitrogen). Treated *L. infantum* promastigotes were incubated with IC_50_ and 2 × IC_50_ of the tested compounds and amphotericin B for 24 h, at 24 °C ± 1 °C. Nontreated parasites were included as negative control and, as positive control, promastigotes were incubated with a mitochondrial membrane potential disrupter, carbonyl cyanide 3-chlorophenylhydrazone (CCCP, 50 μM, Sigma, Portugal). Treated and nontreated parasites were incubated with JC-1 (5,5′,6,6′-tetrachloro 1,1′,3,3′tetraethylbenzimidazol carbocyanine iodide) cationic dye, according to the manufacturer’s instructions. This dye exhibits potential-dependent accumulation in mitochondria, indicated by a fluorescence emission shift from red (~590 nm), JC-1 aggregates to green (~529 nm) JC-1 monomers indicating membrane depolarisation. Briefly, parasites were incubated with JC-1 in PBS (2 μM final concentration) at 24 °C ± 1 °C, for 30 min. Then, parasites were washed once with warm PBS, centrifuged (10 min, 800× *g*) and resuspended in 500 μL PBS. Cells were analysed by flow cytometry. Cells were gated to exclude debris and standard compensation was done with CCCP-treated parasites.

##### Detection of Reactive Oxygen Species

For the detection of reactive oxygen species (ROS), a cell permeable probe CM-H_2_DCFDA (5 µg/mL Molecular Probes, Invitrogen) was used. Briefly, promastigotes were incubated with IC_50_ and 2 × IC_50_ of the tested compounds and amphotericin B for 5 h and 24 h, at 24 °C. Nontreated control and a positive control (H_2_O_2_ 1 mM) were included. Following incubation step, parasites were washed in pre-warmed PBS, centriguged (10 min, 800× *g*) and resuspended in PBS with CM-H_2_DCFDA in a final concentration of 5 μM for 30 min, at 24 °C, and then washed again with PBS. Parasites were resuspended in normal medium (M199 medium) and fluorescence was measured by flow cytometry (blue laser), and the signals registered in the FL1/FITC channel.

#### 4.2.5. Promastigotes DNA Fragmentation

To detect whether the compounds induced fragmentation on *Leishmania* nuclear DNA, promastigotes (1 × 10^7^ parasites/mL) were incubated for 24 h with IC_50_ concentrations of LC132, LC137 and LC138. 2 mM H_2_O_2_ was used as DNA damage inductor and non-treated cells were used as control. After centrifugation (10 min, 800× *g*) DNA was extracted using a commercial extraction kit (High Template DNA Preparation Kit, Roche, Germany), according to the manufacturer’s instructions. Extracted DNA was observed in a 2% (*w/v*) agarose gel at 100 V for 90 min.

#### 4.2.6. Statistical Analysis

Results of IC_50_ and CC_50_ were expressed as the mean ± standard diviation (SD) of three independent experiments. Cell population retrieved from cytometry analysis were expressed as % ± SEM GraphPad Prism 8 (GraphPad Software, Inc.) was used to compare differences among groups in flow cytometry data using two-way ANOVA with Dunnet’s multiple comparisons test using different significance levels (*p* < 0.05, *p* < 0.001, *p* < 0.0001).

## 5. Conclusions

Our findings showed that the selected endoperoxides LC132 and LC138 induce some biochemical and morphological alterations, causing oxidative stress. More biochemical studies are needed to better elucidate the mode of action and the cell death pathway displayed by these compounds. Ultrastrutural alterations of *Leishmania* sp. upon treatment should also be evaluated, in order to have more insigths on compounds targets and mode of action.

LC132 presented better anti-leishmanial intracelular activity on both Old-World *Leishmania* species- *L. infantum* and *L. donovani*- but LC138 showed to be more selective in *L. infantum* when cytotocicity was considered. Upon optimization and pharmacomodulation of these compounds, efficacy evaluation in a VL in vivo model with toxicity evaluation and host immune system involvement, we expect that these class of endoperoxides may furnish promising therapeutic leads against Old-World *Leishmania* parasites worthy of pharmacological consideration.

## Figures and Tables

**Table 1:** CC_50_, IC_50_ and SI of synthetic trioxolanes, amphotericin B and miltefosine, against J774 A.1, THP-1 cell lines and the promastigote forms of L. infantum IMT 151 and L. donovani BD14 after 48 h incubation.

Cytotoxicity			Promastigote’s Susceptibility					
CC_50_ ± SD (µM)			IC_50_ ± SD (µM)					
Compounds	J774A.1 Cell Line	THP-1 Cell Line	*L. infantum*	SI (J774A.1)	SI (THP-1)	*L. donovani*	SI (J774A.1)	SI (THP-1)
LC50	519.3 ± 139.5	712.8 ± 18.4	378.9 ± 2.8	1.4	1.9	353.6 ± 8.4	1.5	2.0
LC93	12.0 ± 0.5	184.9 ± 16.1	435.0 ± 22.7	0.0	0.4	152.9 ± 22.4	0.1	1.2
LC129	323.4 ± 24.5	355.9 ± 31.4	265.9 ± 13.3	1.2	1.3	242,4 ± 10.2	1.3	1.5
LC131	34.1 ± 14.5	78.2 ± 14.1	321.1 ± 11.8	0.1	0.2	123.5 ± 19.0	0.3	0.6
LC132	45.8 ± 19.9	99.8 ± 16.4	13.0 ± 1.7	3.5	7.7	14.3 ± 5.5	3.2	7.0
LC136	113.0 ± 13.8	250.5 ± 4.1	434.2 ± 6.4	0.3	0.6	231.8 ± 28.4	0.5	1.1
LC137	443.2 ± 44.0	3569 ± 22	137.1 ± 0.2	3.2	26.0	143.3 ± 16.5	3.1	24.9
LC138	527.9 ± 54.0	2839 ± 15	125.4 ± 1.6	4.2	22.6	239.6 ± 34.8	2.2	11.9
LC139	912.9 ± 70.7	115.2 ± 11.4	472.3 ± 39.2	1.9	0.2	506.8 ± 53.3	1.8	0.2
LC140	677.6 ± 29.9	1399 ± 76	258.2 ± 6.4	2.6	5.4	460.1 ± 38.3	1.5	3.0
LC146	1465 ± 13	3223 ± 72	491.7 ± 22.7	3.0	6.6	793.0 ± 37.2	1.8	4.1
LC163	474.3 ± 83.5	753.4 ± 90.3	190.3 ± 21.6	2.5	4.0	531.6 ± 105.2	0.9	1.4
AmB	71.9 ± 5.4	23.2 ± 3.0	0.2 ± 0.0	467.7	151.0	0.3 ± 0.0	282.5	91.2
Miltefosine	94.4 ± 17.3	100.4 ± 12.1	148.7 ± 3.5	0.6	0.7	24.2 ± 10.0	3.9	4.1

IC_50_, half-maximal inhibitory concentration; CC_50_, half-maximal cytotoxic concentration; SI, selectivity index; Sis calculated from the ratio between the value of CC_50_ in J774A.1 and THP-1 cells, respectively, and IC_50_ of *Leishmania* spp. promastigotes; AmB, amphotericin B; SD, standard deviation.

**Table 2:** IC_50_ and SI of most active synthetic trioxolanes and control drugs, intracelular amastigote forms of L. infantum IMT151 and L. donovani BD14 after 48 h incubation.

	Amastigote’s Susceptibility IC_50_ ± SD (µM)					
Compounds	*L. infantum*	SI (J774A.1)	SI (THP1)	*L. donovani*	SI (J774A.1)	SI (THP-1)
LC132	13.2 ± 5.2	3.5	7.6	9.4 ± 0.1	4.9	66.3
LC137	168.5 ± 8.9	2.6	21.2	88.3 ± 14.2	5.0	7.5
LC138	23.9 ± 2.7	22.1	118.6	425.9 ± 25.5	1.2	10.6
AmB	0.3 ± 0.1	898.4	298.2	0.1 ± 0.0	898.4	40.4
Miltefosine	16.8 ± 11.7	5.6	6.0	13.3 ± 3.0	7.1	6.7

IC_50_, half-maximal inhibitory concentration; SI, selectivity index; SI calculated from the ratio between the value of CC_50_ in J774A.1 and THP-1 cells, respectively (see Table 1), and IC_50_ of *Leishmania* spp. amastigotes; AmB, amphotericin B; SD, standard deviation.

**Table 3:** Structures of 1,2,4-trioxolanes and 1,2,4,5-tetraoxanes synthesized and concentration ranges employed in cytotoxicity and in vitro activity assays against *Leishmania* spp.

Class	Compounds	Structures	ClogP ^a^	Range of Concentrations (µM)		
				Cytotoxicity assay	Promastigote assay	Intracellular amastigote assay
1,2,4-trioxolanes	LC50	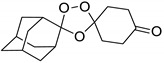	3.06	2156–33.7	538.9–134.7	nd
	LC93	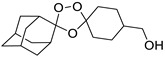	3.30	2038–5.3	679.4–169.8	nd
	LC129	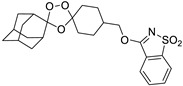	3.86	1306-13.6	326.4–81.6	nd
	LC131	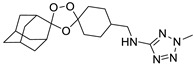	3.81	1599–4.2	266.5–8.3	nd
	LC132	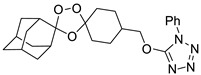	4.05	1368–3.6	228.0–7.2	91.2–2.9
	LC136	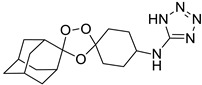	3.03	1728–46.0	576.0–15.3	Nd
1,2,4,5-tetraoxanes	LC137	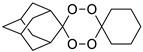	4.19	3569–66.9	356.9–89.2	285.5–8.9
	LC138	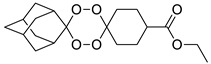	3.91	2839–53.2	567.9–142.0	425.9–13.3
	LC139	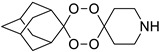	2.12	2134–66.7	711.3–177.8	nd
	LC140	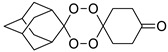	3.04	3400–63.7	679.9–170.0	nd
	LC146	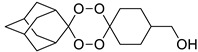	3.10	3224–60.5	967.2–241.8	nd
	LC163	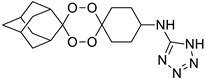	2.94	2753–51.6	826.0–206.5	nd
AmB				118.2–1.7	0.9–0.03	0.4–0.01
Miltefosine				1472–4.6	122.7–3.8	49.0–1.5

^a^ Calculated using ALOGPS software (http://www.vcclab.org/lab/alogps/; accessed 14 November 2021); AmB, amphotericin B; nd, not done.

## Data Availability

Data is contained within the article or supplementary material.

## Supplementary Materials

The following supporting information can be downloaded at: https://www.mdpi.com/article/10.3390/ph15040446/s1. 1. Synthetic procedures and experimental details for the synthesis and chemical characterization of compounds. 1.1. General methods and analytical techniques. 1.2. Preparation of intermediate building blocks. 1.3. Synthetic route to 1,2,4-trioxolanes. 1.4. Synthetic route to 1,2,4,5-tetraoxanes. 2. ^1^H and ^13^C{^1^H} NMR spectra of the synthesized compounds. 3. References.
