# Supplementary material for: 1,2,4-Trioxolane and 1,2,4,5-Tetraoxane Endoperoxides against Old-World Leishmania Parasites: In Vitro Activity and Mode of Action
Source: Pharmaceuticals (Basel). 2022 Apr 3;15(4):446. doi: 10.3390/ph15040446 (PMC9024893; doi:10.3390/ph15040446)
Supplement: Supplementary file 1 [file pharmaceuticals-15-00446-s001.zip › pharmaceuticals-1668157-supplementary.pdf]

Supplementary material

1,2,4-trioxolane and 1,2,4,5-tetraoxane endoperoxides against Old-World *Leishmania* parasites: *in vitro* activity and mode of action

Andreia Mendes[1], Ana Armada[1,2], Lília I.L. Cabral[3,4], Patrícia S.M. Amado[3,4], Ana L. Sousa[5], Erin Tranfield[5], Lenea Campino[1], Maria L.S. Cristiano[3,4]*, Sofia Cortes[1,2]*

[1] Instituto de Higiene e Medicina Tropical (IHMT), Universidade Nova de Lisboa UNL), Rua da Junqueira, 100, 1349-008, Lisboa, Portugal; andricia.mormendes@gmail.com (A.M.); aarmada@ihmt.unl.pt (A.A.); lcampino@ihmt.unl.pt (L.C.); scortes@ihmt.unl.pt (S.C.)

[2] Global Health Tropical Medicine (GHTM), Instituto de Higiene e Medicina Tropical (IHMT), Universidade Nova de Lisboa UNL), Rua da Junqueira, 100, 1349-008, Lisboa, Portugal; aarmada@ihmt.unl.pt (A.A.); scortes@ihmt.unl.pt (S.C.)

[3] CCMAR – Centro de Ciências do Mar, Universidade do Algarve, Campus de Gambelas, 8005-139 Faro, Portugal; liliacabral80@gmail.com (L.I.L.C.); patricia.s.amado@gmail.com (P.S.M.A.); mcristi@ualg.pt (M.L.S.C.)

[4] Departamento de Química e Farmácia, Faculdade de Ciências e Tecnologia, Universidade do Algarve, Campus de Gambelas, 8005-139 Faro, Portugal; liliacabral80@gmail.com (L.I.L.C.); patricia.s.amado@gmail.com (P.S.M.A.); mcristi@ualg.pt (M.L.S.C.)

[5] Electron Microscopy Facility, Instituto Gulbenkian de Ciência, Rua da Quinta Grande, 6, 2780-156 Oeiras, Portugal; alsousa@igc.gulbenkian.pt (A.S.); etranfield@igc.gulbenkian.pt (E.T.)

* Correspondence: mcristi@ualg.pt (M.L.S.C.); scortes@ihmt.unl.pt (S.C.)

Table of contents

**1.**	Synthetic procedures and experimental details for the synthesis and chemical characterization of compounds.	S2
**1.1.**	General methods and analytical techniques	S2
**1.2.**	Preparation of intermediate building blocks	S3
**1.3.**	Synthetic route to 1,2,4-trioxolanes	S4
**1.4.**	Synthetic route to 1,2,4,5-tetraoxanes	S8
**2.**	^{1}H and $^{13}C\{^{1}H\}$ NMR spectra of the synthesised compounds	S12
**3.**	References	S22

1. Synthetic procedures and experimental details for the synthesis and chemical characterization of compounds.

1.1. General methods and analytical techniques

Chemicals. All reagents and solvents used were of analytical grade and were used without further purification. When necessary, solvents were freshly distilled from appropriate drying agents prior to use. Analytical thin layer chromatography (TLC) was carried out using TLC Silica gel 60 F254 aluminium sheets (AL TLC 20x20). Column chromatography was carried out using technical grade Silica Gel 60 (0.04 – 0.063 mm).

Analytical equipment. ^{1}H and ^{13}C Nuclear Magnetic Resonance (NMR) spectra were recorded using a Bruker AMX400 spectrometer or a 500 MHz JEOL system equipped with a Royal HFX probe, in solution, using the deuterated solvents described in each experimental procedure. The chemical shifts (δ) are described in parts per million (ppm) downfield from an internal standard of tetramethylsilane (TMS). High Resolution Mass Spectrometry (HRMS) was recorded using the analytical services within the Centre of Marine Sciences (CCMar). HRMS was on Thermo Scientific High Resolution Mass Spectrometer (HRMS), model Orbitrap Elite, capable of MSn, n up to 10 (CCMar). Thin-layer chromatography was carried out on silica gel 60 F254 plates (AL TLC 20x20). Column chromatography was performed on Silica Gel 60 (0.04 – 0.063 mm). IR spectra were recorded on a Tensor 27 FT/IR spectrometer in the 600–3800 cm^{-1} range. Melting points (°C) were obtained on a SMP30 Melting Point Apparattus and are uncorrected.

Safety. Organic peroxides are potentially hazardous compounds (inflammable and explosive) and must be handled carefully: 1) a safety shield should be used for all reactions involving H_2O_2; 2) direct exposure to strong heat or light, mechanical shock, oxidizable organic materials or transition-metal ions should be avoided.

1.2. Preparation of intermediate building blocks

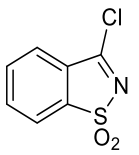

3-chloro-1,2-benzisothiazole-1,1-dioxide

Procedure followed by Ismael *et al.*[1] Starting from saccharin (56 mmol) and phosphorus pentachloride (66 mmol), heated at 200 ºC. Colourless needles from ethanol (63% yield); m.p. 143-145 ºC. IR ν_{max} (cm^{-1}): 1724, 1654, 1603 (C=C), 1346 (SO_2), 775 (Ar-H) and 692 (C-Cl); ^{1}H-NMR (400 MHz, $CDCl_3$-*d*): δ 7.85 (4H, m, Ar-H) ppm. Found: C, 41.5%; H, 2.0%; N, 6.9%; calcd for $C_7H_4NO_2SCl$: C, 41.7%; H, 2.0%; N, 7.0%. MS (EI, *m*/*z*): 201 [M]$^+$.

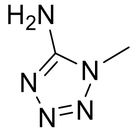

1-methyl-*1H*-tetrazole-5-amine, LC126I

A solution of sodium hydroxide (20%) was added dropwise to a suspension of 5-aminotetrazole monohydrate (120 mmol) in water (30 mL), with a drop of phenolphthalein. The mixture was stirred until complete dissolution of the suspended material. Dimethyl sulphate (110 mmol) was then added in small portions, keeping an alkaline medium through addition of aqueous sodium hydroxide. The final mixture was refluxed for 1 h, then cooled, and finally left in ice bath for 48h. Colourless needles of the desired compound were filtered and dried (51% yield); m.p. 220-221ºC. ^{1}H-NMR (400 MHz, $CDCl_3$-*d*): δ 4.15 (s, 3H) ppm; MS (EI, *m*/*z*): 99 [M]$^+$.

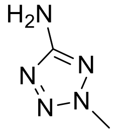

2-methyl-*2H*-tetrazole-5-amine, LC126II

The filtrate from 1-methyl-1H-tetrazole-5-amine **LC126I** synthesis was evaporated under reduced pressure to afford a solid residue. Water (50 mL) was added, and the mixture was then extracted with diethyl ether (3 x 50 mL). The organic extract was dried over anhydrous sodium sulfate, filtered, and the filtrate evaporated to afford colourless crystals. Recrystallization from diethyl ether gave the desired compound as colourless needles (25% yield); m.p. 104.5-105.5ºC. ^{1}H-NMR (400 MHz, $CDCl_3$-*d*): δ 3.32 (s, 3H) ppm; MS (EI, *m*/*z*): 99 [M]$^+$.

1.3. Synthetic route to 1,2,4-trioxolanes

The synthetic approach followed to 1,2,4-trioxolanes is illustrated in **Scheme S1.** Synthetic procedures for the preparation of each compound are also provided in this section.

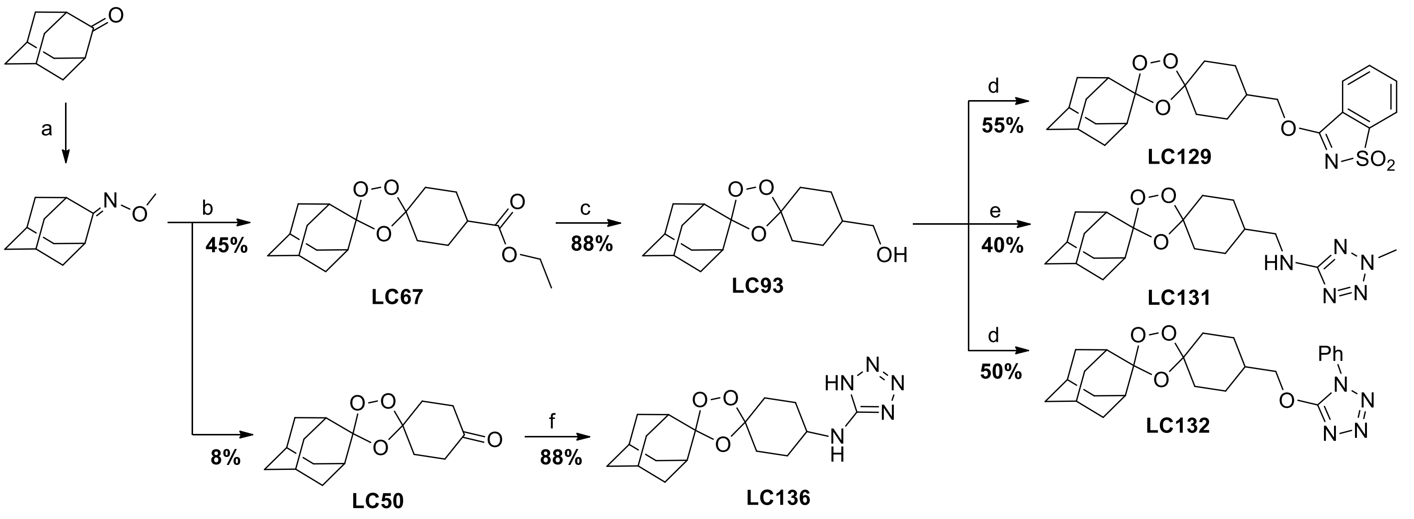

Scheme S1: Reagents and conditions: a) Pyridine, $MeONH_2$, MeOH, r.t; b) ketone, O_3, DCM/Pentane, -78ºC; c) 1. $LiAlH_4$, anhydrous Et_2O, 0ºC, 1h; 2. H_2O; d) Chloride derivative, TEA, Toluene, 45ºC; e) 1. Triethyamine, mesyl chloride, THF, 3h, 2. Amine, 60ºC; f) 2-methyl-2*H*-tetrazole-5-amine, AcOH, DCE, $NaBH(OAc)_3$, r.t.

General procedure 1: Preparation of the 1,2,4-trioxolane ring. Trioxolanes were prepared by coupling O-methyl-2-adamantanone oxime (2) with a cyclohexanone derivative, through ozonolysis. Ozone, produced with an ozone generator Sander Labor-Ozonizator 301.7 (0.5 L/min O_2, 140 V), was passed through a solution of dichloromethane at –78ºC and flushed into a solution of O-methyl ketone oxime and a ketone, in pentane/dichloromethane (6:4) at 0ºC. After completion, the solution was flushed with nitrogen for 5 min and concentrated under reduced pressure at room temperature to give a crude material that was purified by column chromatography.

General Procedure 2: Reduction with $LiAlH_4$. Procedure followed by Kwiatkowski and *et al.*[2] with slight modifications. To a suspension of $LiAlH_4$ (3 mmol) in anhydrous ether at 0ºC (5 mL) was added dropwise, the corresponding carboxylic acid or ester (1 mmol) in diethyl ether (5 mL). The reaction mixture was stirred at 0 °C for 60 minutes. The mixture reaction was then quenched successively with H_2O (2 mL) and 6M NaOH (1.0 mL) and it was allowed to warm to ambient temperature while stirring. Anhydrous Na_2SO_4 (2g) was added, stirred for 30 minutes, filtered over a pad of celite and washed with EtOAc (3x15 mL). The combined orgainc layers were concentrated under reduced pressure to afford the desired product.

General Procedure 3: Nucleophilic substitution with chloride derivatives. Procedure followed by Lobo *et al.*[3] The corresponding chloride substituted compound (1.2 mmol) was added to a solution of compound the peroxide alcohol

(1 mmol) in dry toluene (10 mL). The solution was stirred at 45 ºC for 15 minutes, followed by addition of triethylamine (2 mmol) until disappearance of all starting material. The precipitate of triethylamine hydrochloride was filtered off and the filtrate was evaporated to give a yellow crystalline solid, which was recrystallized from ethanol.

General Procedure 4: Nucleophilic substitution with amines. Procedure followed by Lobo *et al.*[3] To a solution of peroxide alcohol (1 mmol) in THF (10 mL) was added mesyl chloride (1.1 mmol) and triethylamine (2 mmol). The solution was stirred at room temperature for 3 hours. Then a solution of the corresponding amine (1.5 mmol) in THF (10 mL) was added dropwise to the stirred suspension, over 30 minutes. The mixture was stirred at 65 ºC for 24 hours. Excess solvent was then removed. Recrystallization from ethanol gave the desired product.

General Procedure 5: Reductive amination. Procedure followed by Lobo *et al.*[3] The corresponding crude aldehyde (1 mmol) and the corresponding amine (1.5 mmol) were dissolved in dichloroethane (DCE, 10 mL), and it was added glacial acetic acid (1.1 mmol). The mixture was allowed to stir at room temperature for 30 minutes followed by addition of sodium triacetoxyborohydride (2.5 mmol) which was left to stir for 16 h. hours. The reaction mixture was washed with aqueous NaOH (5M; 2 x 10 mL) and dichloromethane (2 x 20 mL). The combined organic layers were dried over $MgSO_4$, filtered, and the solvent removed under reduced pressure. The residue was purified by flash chromatography using a 0–40% EtOAc–hexane gradient (unless specified differently) to obtain desired product.

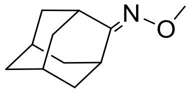

2-(Methoxyimino)adamantane

To a solution of 2-adamantanone (30 mmol) in methanol (30 mL) were added pyridine (55.6 mmol) and methoxylamine hydrochloride (45.0 mmol). The reaction mixture was stirred at room temperature for 48 h. The final mixture was concentrated and then diluted with DCM (50 mL) and water (50 mL). The organic layer was separated and the aqueous layer was extracted with DCM (30 mL). The combined organic extracts were washed with aqueous HCl (1 M; 30 mL x2), then with saturated aqueous NaCl (30 mL). The final organic extract was dried over $MgSO_4$, filtered and concentrated under reduced pressure to give O-methyl-2-adamantanone oxime (89% yield) as a colourless solid. M.p. 69-70ºC (Lit.[4] 70-71ºC). ^{1}H NMR (400 MHz, $CDCl_3$-*d*): δ 1.69-2.02 (m, 14H), 2.14 (t, J = 6.9 Hz, 4H), 2.51 (t, J = 7.0 Hz, 4H) ppm; $^{13}C\{^{1}H\}$ NMR (100 MHz, $CDCl_3$-*d*): 25.9, 26.31, 31.09, 32.59, 34.25, 35.70, 36.18, 37.35, 106.46, 111.95, 208.90 ppm. MS (MALDI-TOF, *m/z*): 180.02 $[M]^+$.

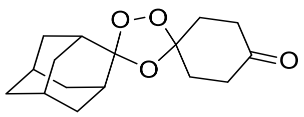

Dispiro[cyclohexane-1,3'-[1,2,4]trioxolane-5',2''-tricyclo[3.3.1.1^{3,7}]decan]-4-one (LC50).

This compound was synthesised in accordance with general procedure 1 using O-methyl 2-adamantanone oxime and 2-adamantanone. Purification by flash chromatography (EtOAc: n-hexane, 10:90, v/v) provided a colourless solid (42% yield). M.p. 127-128ºC (Lit.[3] 126-128ºC). Spectral data are in accordance with the reported in the literature.[3] ^{1}H-NMR (400 MHz, $CDCl_3$-*d*): δ 1.69-2.02 (m, 14H), 2.14 (t, *J* = 7,1 Hz, 4H), 2.51 (t, *J* = 7,1 Hz, 4H) ppm; ^{13}C{^{1}H} NMR (100 MHz, $CDCl_3$-*d*): 25.9, 26.31, 31.09, 32.59, 34.25, 35.70, 36.18, 37.35, 106.46, 111.95, 208.90 ppm; MS (ESI+, *m*/*z*): 278.9 [M + H]$^{+}$.

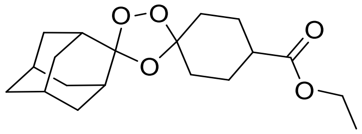

Ethyl dispiro[cyclohexane-1,3'-[1,2,4]trioxolane-5',2''-tricyclo[3.3.1.1^{3,7}]decane]-4-carboxylate (LC67).

This compound was synthesised in accordance with general procedure 1 using O-methyl 2-adamantanone oxime and ethyl 4-oxocyclohexanecarboxylate. Purification by flash chromatography (EtOAc: n-hexane, 5:95, v/v) provided a colourless oil (46% yield). ^{1}H NMR (500 MHz, $CDCl_3$-*d*): δ 4.11 (dq, *J* = 9.4, 7.1 Hz, 2H), 2.40 – 2.26 (m, 1H), 2.00 – 1.66 (m, 22H), 1.24 (dt, *J* = 8.3, 7.1 Hz, 3H) ppm. ^{13}C{^{1}H} NMR (126 MHz, $CDCl_3$-*d*): δ 175.04, 111.60, 108.04, 60.49, 41.52, 41.30, 37.15, 36.85, 36.44, 34.94, 34.90, 34.85, 34.83, 34.06, 33.66, 33.44, 33.29, 33.23, 31.59, 27.17, 27.02, 26.94, 26.53, 26.31, 26.13, 14.33, 14.30 ppm. Duplicate peaks on ^{13}C{^{1}H} NMR is due to the mixture of isomers ***cis*** or ***trans***. MS (MALDI-TOF, *m*/*z*): 337.34 [M]$^{+}$.

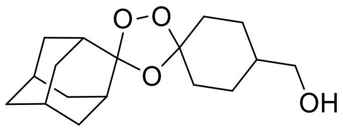

Dispiro[cyclohexane-1,3'-[1,2,4]trioxolane-5',2''-tricyclo[3.3.1.1^{3,7}]decan]-4-yl)methanol (LC93).

This compound was synthesised in accordance with general procedure 2 using ethyl dispiro[cyclohexane-1,3'-[1,2,4]trioxolane-5',2''-tricyclo[3.3.1.1^{3,7}]decane]-4-carboxylate (**LC67**). Yellow crystalline solid (90% Yield). M.p. 99-101ºC. Spectral data are in accordance with the reported in the literature.[3] ^{1}H NMR (500 MHz, $CDCl_3$-*d*): δ 3.53 – 3.45 (m, 2H), 2.12 – 1.65 (m, 21H), 1.58 – 1.15 (m, 5H) ppm. ^{13}C{^{1}H} NMR (126 MHz, $CDCl_3$-*d*): δ 111.43, 109.00, 67.75, 39.19, 38.94, 36.87, 36.59, 36.46, 34.99, 34.89, 34.87, 34.82, 34.62, 33.88, 33.69, 33.29, 31.10, 27.17, 26.97, 26.95, 26.70, 26.55, 26.53 ppm. Duplicate peaks on ^{13}C {^{1}H} NMR is due to the mixture of isomers ***cis*** or ***trans***. MS (MALDI-TOF, *m*/*z*): 318.30 [M + Na]$^{+}$.

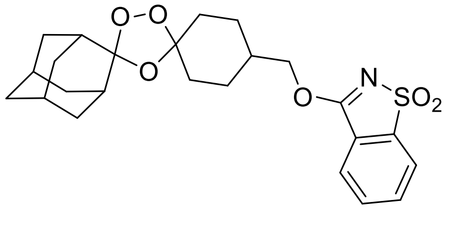

3-{(Dispiro[cyclohexane-1,3'-[1,2,4]trioxolane-5',2''-tricyclo[3.3.1.1^{3,7}]decan]-4-yl)methoxy}-*1H*-1λ^{6},2-benzisothiazole-1,1-dione (LC129)

This compound was synthesised in accordance with general procedure 3 using dispiro[cyclohexane-1,3'-[1,2,4]trioxolane-5',2''-tricyclo[3.3.1.1^{3,7}]decan]-4-yl)methanol (**LC93**) and 3-chloro-1,2-benzisothiazole-1,1-dioxide. Recrystallization from ethanol gave the desired product as yellow crystalline solid (50% yield). M.p. 150-151ºC. ^{1}H-NMR (400 MHz, $CDCl_3$-*d*): δ 1.35-1.38 (m, 2H), 1.62-1.96 (m, 21H), 4.36 (d, *J* = 7.2 Hz, 2H), 7.64 (d, *J* = 7.1 Hz, 1H), 7.69 (d, *J* = 7.0 Hz, 2H), 7.82 (d, *J* = 7.2 Hz, 1H) ppm. ^{13}C{^{1}H} NMR (100 MHz, $CDCl_3$-*d*): 17.60, 26.87, 29.23, 32.36, 35.39, 37.95, 39.14, 66.39, 108.22, 117.62, 123.30, 127.03, 133.44, 134.12, 143.61, 169.23 ppm. MS (ESI+, *m/z*): 482.25 [M + Na]$^{+}$. Anal. calcd for $C_{24}H_{29}NO_6S$: C, 62.73%; H, 6.36%; N, 3.05%; Found: C, 62.85%; H, 6.44%; N, 2.99%.

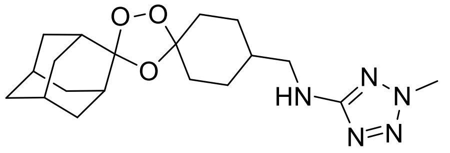

{(Dispiro[cyclohexane-1,3'-[1,2,4]trioxolane-5',2''-tricyclo[3.3.1.1^{3,7}]decan]-4-yl)methyl}(2-methyl-2*H*-1,2,3,4-tetraazol-5-yl)amine (LC131)

This compound was synthesised in accordance with general procedure 4 using dispiro[cyclohexane-1,3'-[1,2,4]trioxolane-5',2''-tricyclo[3.3.1.1^{3,7}]decan]-4-yl)methanol (**LC93**) and 2-methyl-*2H*-tetrazole-5-amine, (**LC126II**). Purification by flash chromatography (EtOAc: *n*-hexane, 20:80, v/v) provided a yellow solid (75% yield. M.p. 110-112 ºC. Spectral data are in accordance with the reported in the literature.[3] ^{1}H-NMR (400 MHz, $CDCl_3$-*d*): 3.66 (s, 3H), 2.93 (m, 2H), 1.61-1.92 (m, 16H), 1.24-1.47 (m, 4H), 1.16-1,19 (m, 3H) ppm. MS (MALDI-TOF, *m/z*): 415.15 [M+K]$^{+}$.

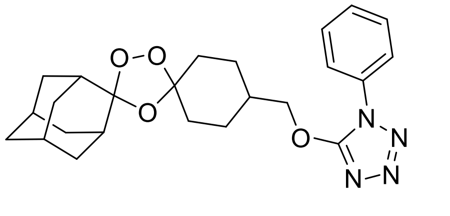

5-{(Dispiro[cyclohexane-1,3'-[1,2,4]trioxolane-5',2''-tricyclo[3.3.1.1^{3,7}]decan]-4-yl)methoxy}-1-phenyl-1*H*-1,2,3,4-tetraazole (LC132)

This compound was synthesised in accordance with general procedure 3 using dispiro[cyclohexane-1,3'-[1,2,4]trioxolane-5',2''-tricyclo[3.3.1.1^{3,7}]decan]-4-yl)methanol (**LC93**) and 5-chloro-1-phenyl-1*H*-tetrazole. Recrystallization from ethanol gave LC132 as a white solid (32% yield). M.p. 82-84 ºC. Spectral data are in accordance

with the reported in the literature.[3] ^{1}H-NMR (400 MHz, $CDCl_3$-*d*): 7.73 (d, 2H), 7.57 (t, 2H), 7.48 (t, 1H), 4.50 (d, 2H), 2.01 (d, 7H), 1.71-1.86 (m, 13H), 1.39-1.46 (m, 2H), 1.27 (s, 1H) ppm; MS (MALDI-TOF, *m*/*z*): 477.11 $[M+K]^+$.

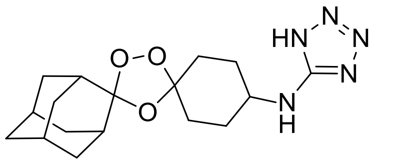

***N-1H*-1,2,3,4-Tetraazol-5-yl-dispiro[cyclohexane-1,3'-[1,2,4]trioxolane-5',2''-tricyclo[3.3.1.1^{3,7}]decan]-4-ylamine (LC136)**

This compound was synthesised in accordance with general procedure 5 using dispiro[cyclohexane-1,3'-[1,2,4]trioxolane-5',2''-tricyclo[3.3.1.1^{3,7}]decan]-4-one (**LC50**) and 5-aminotetrazole. Purification by flash chromatography (EtOAc: n-hexane, 15:85, v/v) provided a white solid (80% yield). M.p 98-100ºC. Spectral data are in accordance with the reported in the literature.[3] ^{1}H-NMR (400 MHz, $CDCl_3$-*d*): δ 1.24-1.33 (m, 2H), 1.69-1.72 (m, 10H), 1.90-2.05 (m, 10H), 2.66 (m, 1H) ppm. ^{13}C {^{1}H} NMR (100 MHz, $CDCl_3$-*d*): 21.05, 27.07, 28.42, 33.67, 36.78, 37.64, 58.57, 117.87, 127.78, 156.15 ppm; MS (MALDI-TOF, *m*/*z*): 347.31 $[M + H]^+$.

1.4. Synthetic route to 1,2,4,5-tetraoxanes

The synthetic approach followed to 1,2,4,5-tetraoxanes is depicted in **Scheme S2**. Synthetic procedures for each compound prepared are also provided in this section.

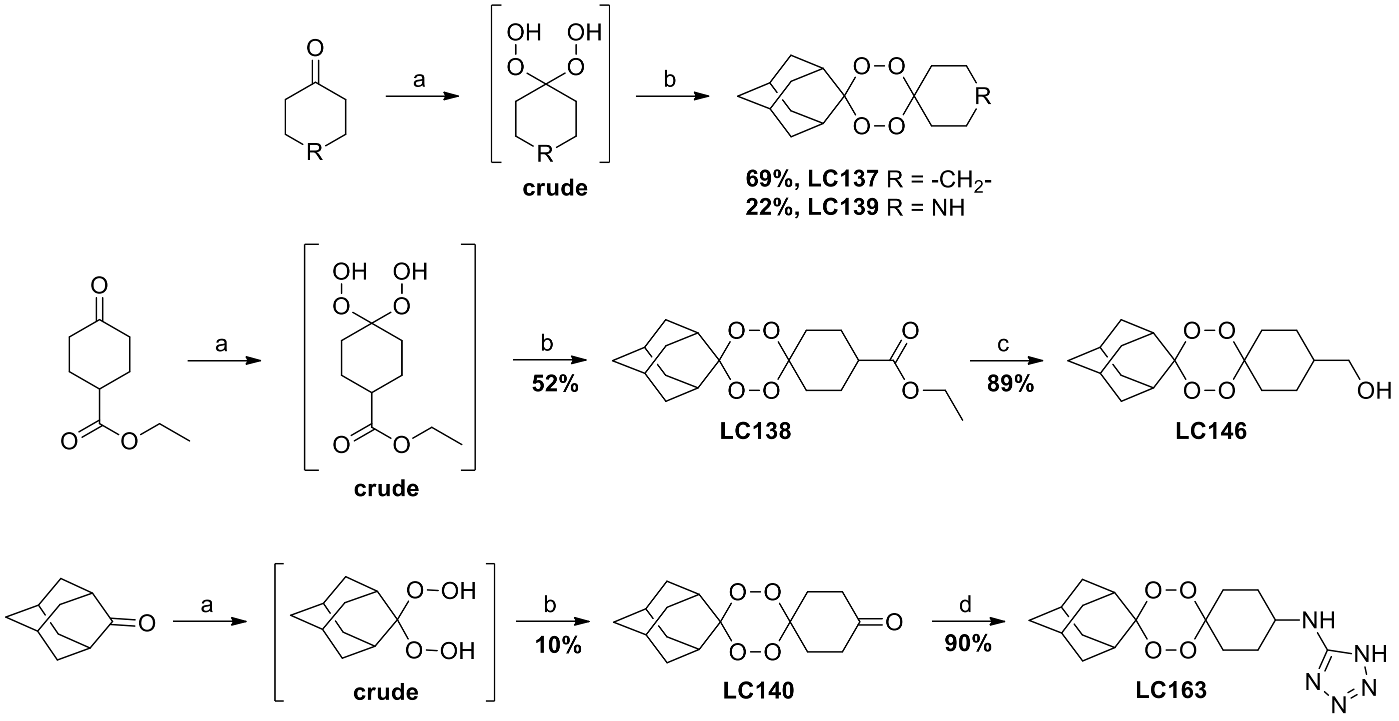

Scheme S2: Reagents and conditions: a) SSA, CH_3CN, H_2O_2 50% (w/w), 0ºC-rt; b) Ketone, SSA (2 eq), anhydrous CH_2Cl_2, rt, 2 h; c) 1. $LiAlH_4$, anhydrous Et_2O, 0ºC, 1h; 2. H_2O; d) 5-Aminotetrazole, DCE, AcOH, $NaBH(OAc)_3$, rt.

General Procedure 6: Synthesis of 1,2,4,5-Tetraoxanes. Procedure followed by Amado *et al.*[4] ***Step 1:*** Carbonyl compound **1** (1 mmol) was dissolved in acetonitrile (3 mL) and **SSA-(C)** (2 mmol) was added to the mixture. Hydrogen peroxide 50 wt. % in H_2O (4 mmol) was slowly added, over an ice bath, then the mixture was left to stir at room temperature until consumption of the starting material. To this mixture was added distilled water, then the catalyst was filtered and rinsed with CH_2Cl_2. The filtrate was extracted with CH_2Cl_2 (3 × 30 mL), dried over with $MgSO_4$, and concentrated under reduced pressure, at low temperature (30-35°C), to obtain the *gem*-dihydroperoxide semi-crude, which was used immediately, without further purification. ***Step 2:*** The *gem*-dihydroperoxide semi-crude was dissolved in anhydrous CH_2Cl_2 (5 mL), followed by addition of the second carbonyl compound **2** (1.5 mmol). The mixture was cooled over an ice bath, prior to addition of **SSA** (2 mmol). The mixture was then warmed and left to stir at room temperature until consumption of the starting material. The resulting solution was then filtered, rinsed with CH_2Cl_2, and concentrated under reduced pressure. The residue was purified by flash chromatography using an EtOAc−hexane gradient (unless specified differently) to afford pure 1,2,4,5-tetraoxanes. *Procedure for preparation of silica sulfuric acid (SSA).* To a slurry of silica gel (10 g, 230–400 mesh, pore size 60 Å) in dry diethyl ether (50 mL) was added concentrated H_2SO_4 (>95%, 3 mL) under strong stirring, for 30 min, at 0°C. The solvent was evaporated under reduced pressure, resulting in free-flowing silica sulfuric acid that was dried *in vacuo* for 24 hours. Then, it was heated at 120°C for 3 h (using a hot plate), affording the catalyst **SSA-(C).** The prepared catalyst was stored inside in a desiccator.

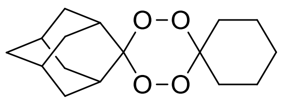

Dispiro[cyclohexane-1,3'-[1,2,4,5]tetraoxane-6',2''-tricyclo[3.3.1.1^{3,7}]decane] (LC137)

This compound was synthesised in accordance with general procedure 6 using cyclohexanone (for the peroxidation step) and 2-adamantaone (cyclocondensation step). Purification by flash chromatography (*n*-hexane, 100%, v/v) provided a white solid (179 mg, 64% yield). M.p = 57-59ºC. ^{1}H NMR (500 MHz, $CDCl_3$-*d*): δ 3.17 (s, 1H), 2.30 (s, 2H), 2.04 – 1.44 (m, 21H). $^{13}C\{^1H\}$ NMR (126 MHz, $CDCl_3$-*d*): δ 110.4, 108.1, 37.1, 37.1, 34.4, 33.3, 33.2, 31.9, 30.2, 29.7, 27.2, 25.5, 22.4. HRMS (MALDI-TOF, *m/z*) calcd for $C_{16}H_{24}O_4K$ $(M+K)^+$: 318,1311; found 318.3302.

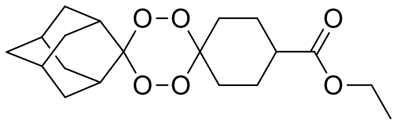

Ethyl dispiro [cyclohexane-1,3'-[1,2,4,5]tetraoxane-6',2''-tricyclo[3.3.1.1^{3,7}]decane]-4-carboxylate (LC138)

This compound was synthesised in accordance with general procedure 6 using 2-ethyl 4-oxocyclohexanecarboxylate (for the peroxidation step) and 2-adamantanone (for the cyclocondensation step). Purification by flash chromatography (EtOAc: *n*-hexane, 1:99, v/v) provided a white solid (49% yield). M.p. = 67-69°C. ^{1}H NMR (500 MHz, $CDCl_3$-*d*): 4.12 (q, *J* = 7.1 Hz, 2H), 3.02 (br d, *J* = 118.6 Hz, 2H), 2.41 – 2.34 (m, 1H), 2.08 – 1.60 (m, 19H), 1.50 (s, 1H), 1.23 (t, *J* = 7.1 Hz, 3H).

$^{13}C\{^1H\}$ NMR (126 MHz, $CDCl_3$-*d*): δ 174.8, 110.6, 107.3, 60.5, 41.8, 39.4, 37.0, 34.4, 33.2, 30.2, 30.2, 28.3, 27.1, 24.8, 23.9, 14.3. HRMS (ESI$^+$, *m*/*z*) calcd $C_{19}H_{28}O_6Na$ (M+Na)$^+$: 375.17781; found 375.17725.

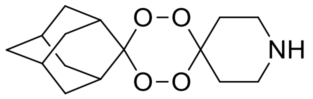

Dispiro[piperidine-4,3'-[1,2,4,5]tetraoxane-6',2''-tricyclo[3.3.1.1^{3,7}]decane] (LC139)

This compound was synthesised in accordance with general procedure 6 using 4-piperidone (for the peroxidation step) and 2-adamantanone (for the cyclocondensation step). Purification by flash chromatography (EtOAc: *n*-hexane, 40:60, v/v) provided a yellow solid (51% yield); M.p. 65-67ºC. ^{1}H-NMR (400 MHz, $CDCl_3$-*d*): δ 1.26 (s, 1H), 1.75 (d, *J* = 7.3 Hz, 6H), 2.04 (m, 14H), 3.08 (t, *J* = 7.1 Hz, 2H) ppm; $^{13}C\{^1H\}$-NMR (100 MHz, $CDCl_3$-*d*): 27.80, 29.67, 32.67, 33.81, 36.68, 107.39, 109.46 ppm; MS (MALDI-TOF, *m*/*z*): 282.29 [M+H]$^+$.

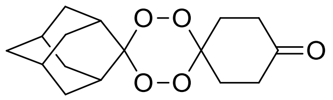

Dispiro[cyclohexane-1,3'-[1,2,4,5]tetraoxane-6',2''-tricyclo[3.3.1.1^{3,7}]decan]-4-one (LC140)

This compound was synthesised in accordance with general procedure 6 using 2-adamantanone (for the peroxidation step) and 1,4-cyclohexanedione (for the cyclocondensation step). Purification by flash chromatography (EtOAc: *n*-hexane, 2.5:97.5, v/v) provided a white solid (8% yield). M.p = 156-158ºC. ^{1}H NMR (500 MHz, $CDCl_3$-*d*): δ 3.20 (br s, 1H), 2.72 (s, 2H), 2.48 (br d, 4H), 2.10 – 1.86 (m, 9H), 1.82 – 1.59 (m, 6H). $^{13}C\{^1H\}$ NMR (126 MHz, $CDCl_3$-*d*): δ 209.4, 111.1, 106.7, 37.0, 36.5, 35.7, 34.4, 33.2, 30.5, 30.2, 28.0, 27.1. HRMS (ESI$^+$, *m*/*z*) calcd $C_{16}H_{22}O_5Na$ (M+Na)$^+$: 317.13594; found 317.13599.

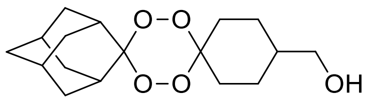

(Dispiro[cyclohexane-1,3'-[1,2,4,5]tetraoxane-6',2''-tricyclo[3.3.1.1^{3,7}]decan]-4-yl)methanol (LC146)

This compound was synthesised in accordance with general procedure 2 using ethyl dispiro[cyclohexane-1,3'-[1,2,4,5]tetraoxane-6',2''-tricyclo[3.3.1.1^{3,7}]decane]-4-carboxylate (**LC138**). White solid (89% yield). M.p. 175-177ºC. Spectral data are in accordance with the reported in the literature.[3] ^{1}H-NMR (400 MHz, $CDCl_3$-*d*): δ 1.19 (m, 2H), 1.64 (s, 6H), 1.69-1.93 (m, 13H), 2.14 (d, *J* = 6.7 Hz, 2H), 3.47 (s, 2H) ppm; $^{13}C\{^1H\}$ NMR (100 MHz, $CDCl_3$-*d*): 26.57, 26.78, 27.14, 33.64, 34.91, 34.99, 36.59, 36.98, 39.03, 67.72, 108.68, 111.59 ppm; MS (MALDI-TOF, *m*/*z*): 309.35 [M]$^-$.

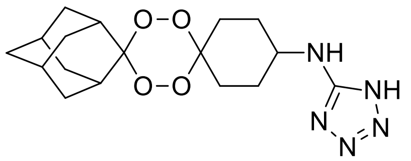

***N-1H*-1,2,3,4-Tetraazol-5-yl-dispiro[cyclohexane-1,3'-[1,2,4,5]tetraoxane-6',2''-tricyclo[3.3.1.1^{3,7}]-decan]-4-ylamine (LC163)**

This compound was synthesised in accordance with general procedure 5 using dispiro[cyclohexane-1,3'-[1,2,4,5]tetraoxane-6',2''-tricyclo[3.3.1.1^{3,7}]decan]-4-one **(LC140)** and 5-aminotetrazole. Purification by flash chromatography (EtOAc: n-hexane, 15:15, v/v) provided a white solid (95% yield). M.p. 142-144ºC. ^{1}H-NMR (400 MHz, $CDCl_3$-*d*): δ 1.15-1.22 (m, 2H), 1.60-1.70 (m, 10H), 1.80-2.05 (m, 10H), 2.6 (m, 1H) ppm; ^{13}C{^{1}H} NMR (100 MHz, $CDCl_3$-*d*): 20.05, 26.06, 27.42, 29.67, 32.78, 33.64, 59.57, 106.44, 111.91 ppm; MS (MALDI-TOF, *m*/*z*): 363.43 $[M]^{+}$.

2. ^{1}H and ^{13}C{^{1}H} NMR spectra of the synthesised compounds

^{1}H NMR spectrum (400 MHz) of **2-(Methoxyimino)adamantane** in $CDCl_3$-*d*.

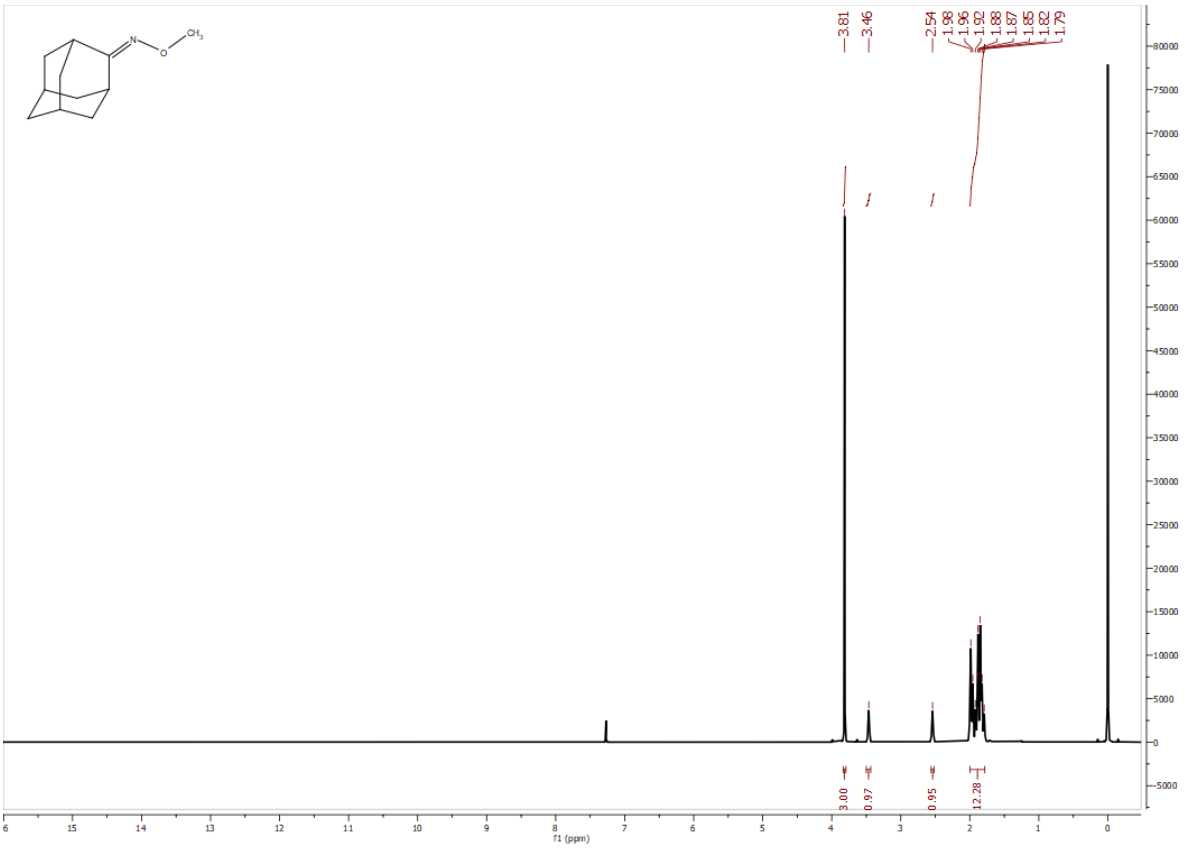

^{13}C{^{1}H} NMR spectrum (100 MHz) of **2-(Methoxyimino)adamantane** in $CDCl_3$-*d*.

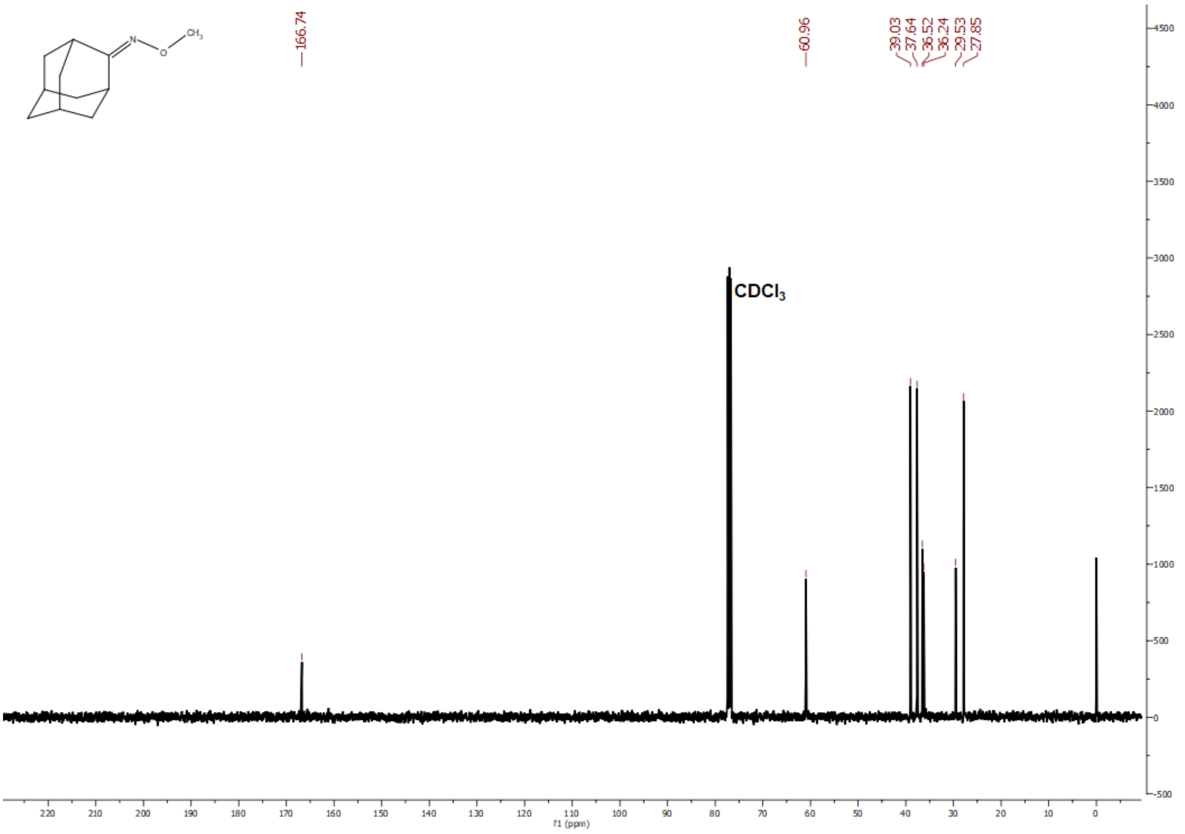

^{1}H NMR (500 MHz, $CDCl_3$) spectrum of compound **LC67**

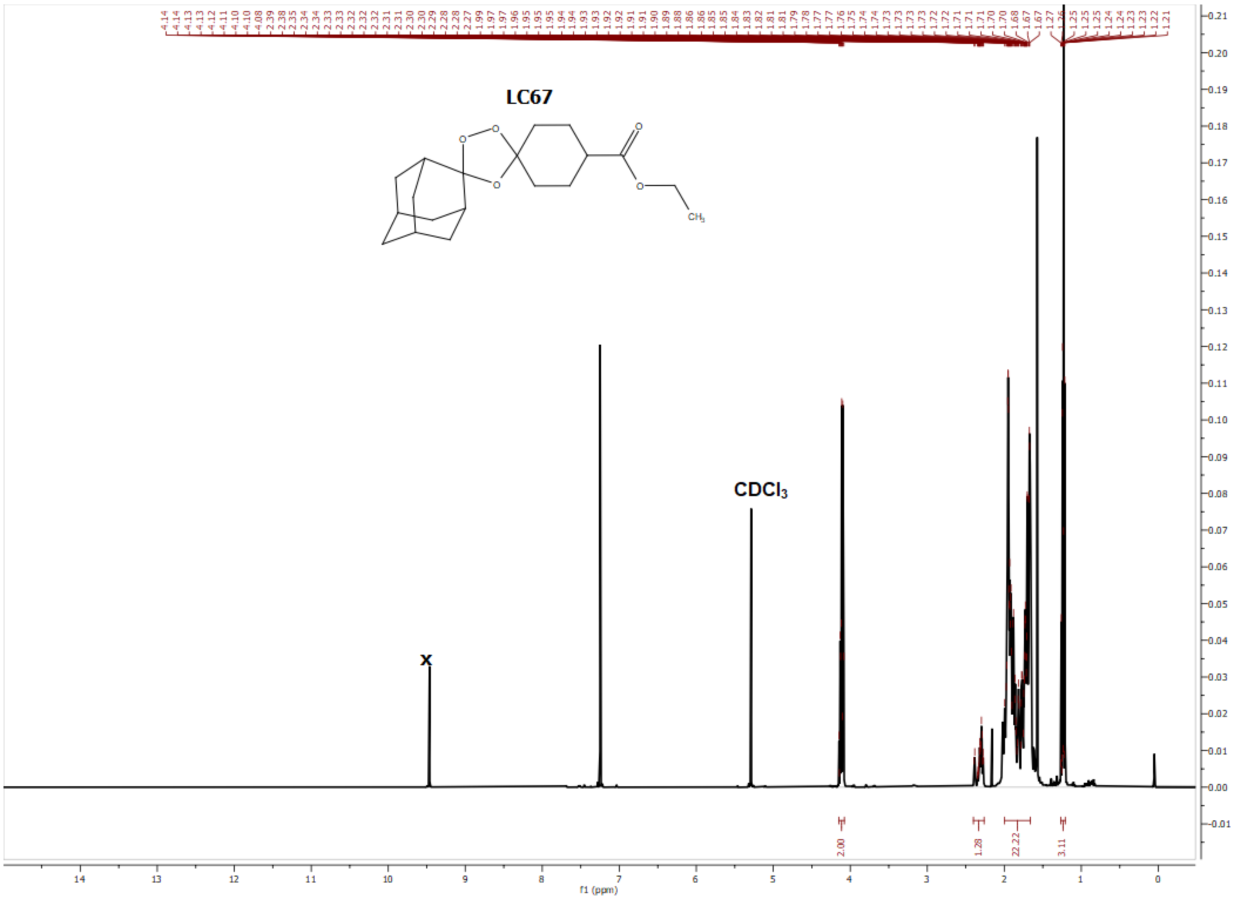

^{13}C {^{1}H} NMR (126 MHz, $CDCl_3$) spectrum of compound **LC67**

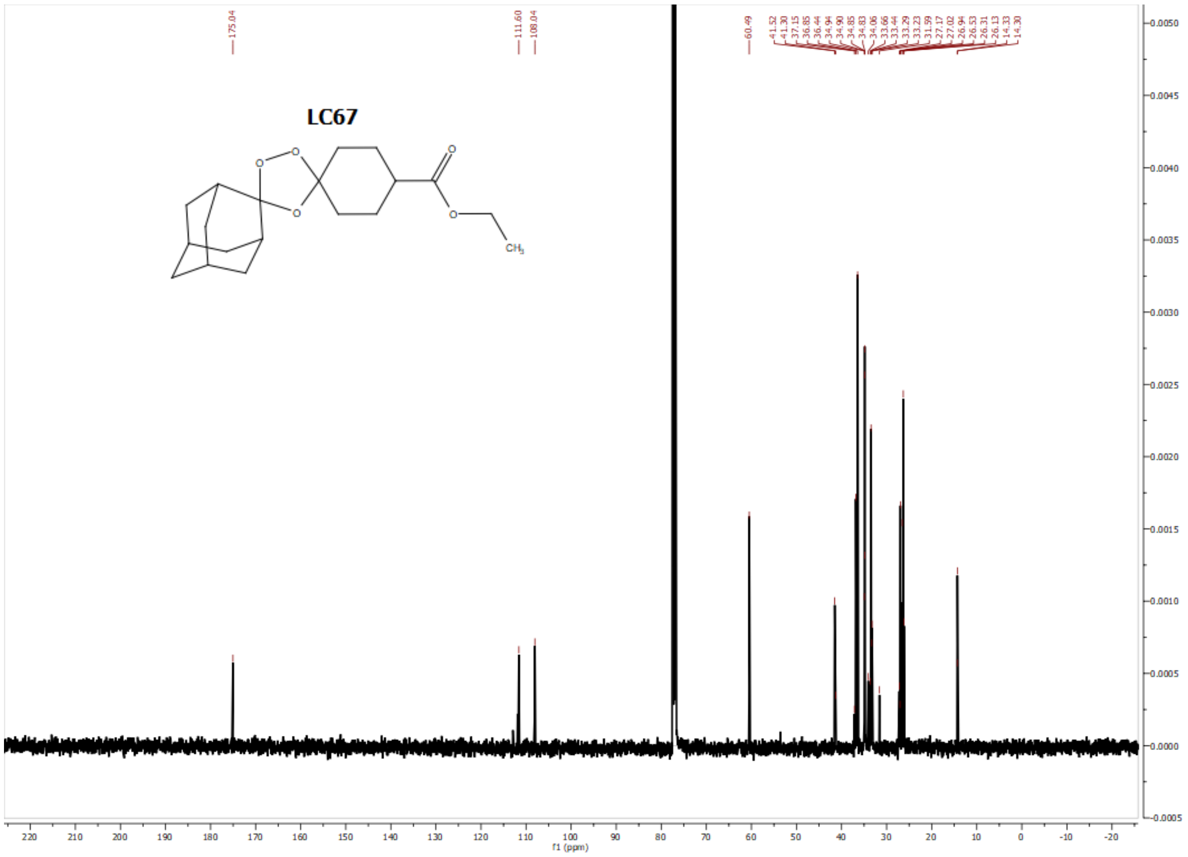

^{1}H NMR (500 MHz, $CDCl_3$) spectrum of compound **LC93**

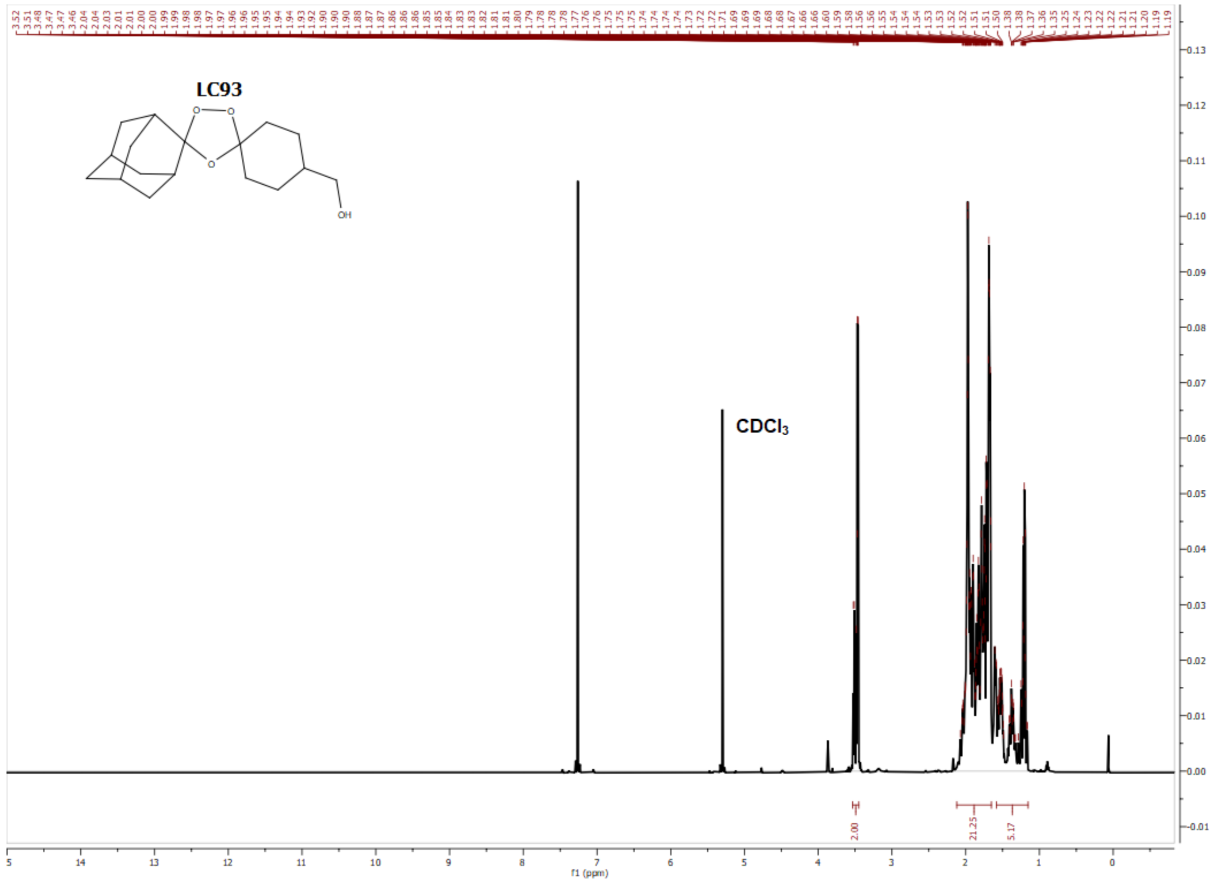

^{13}C {^{1}H} NMR (126 MHz, $CDCl_3$) spectrum of compound **LC93**

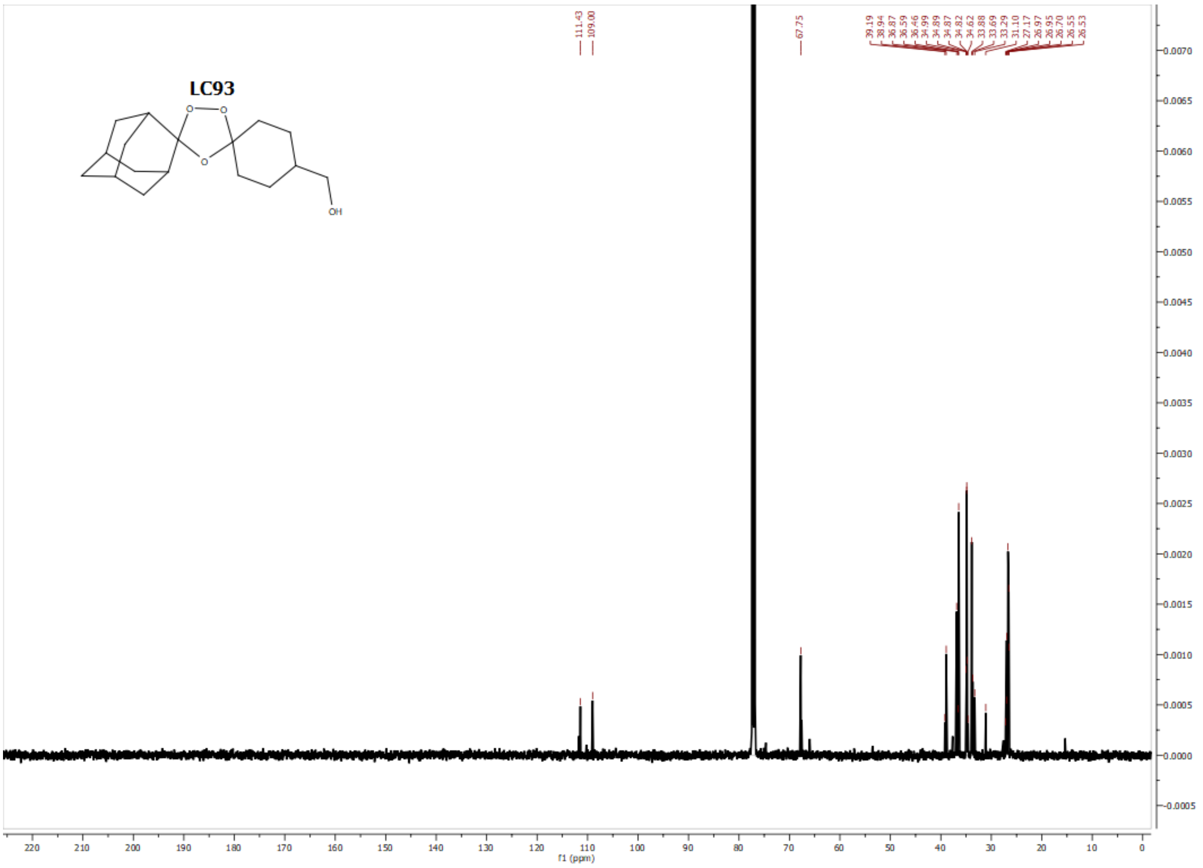

^{1}H NMR (400 MHz, $CDCl_3$) spectrum of compound **LC129**

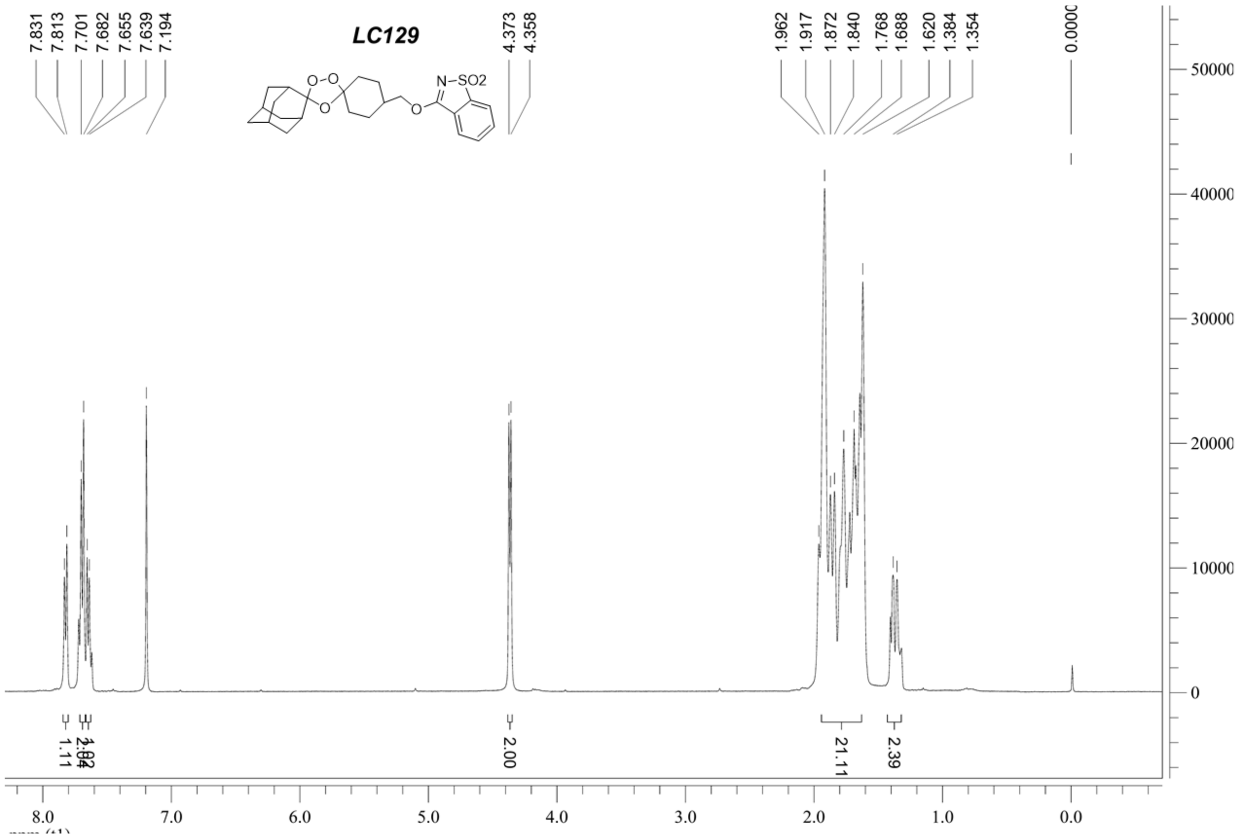

^{13}C {^{1}H} NMR (100 MHz, $CDCl_3$) spectrum of compound **LC129**

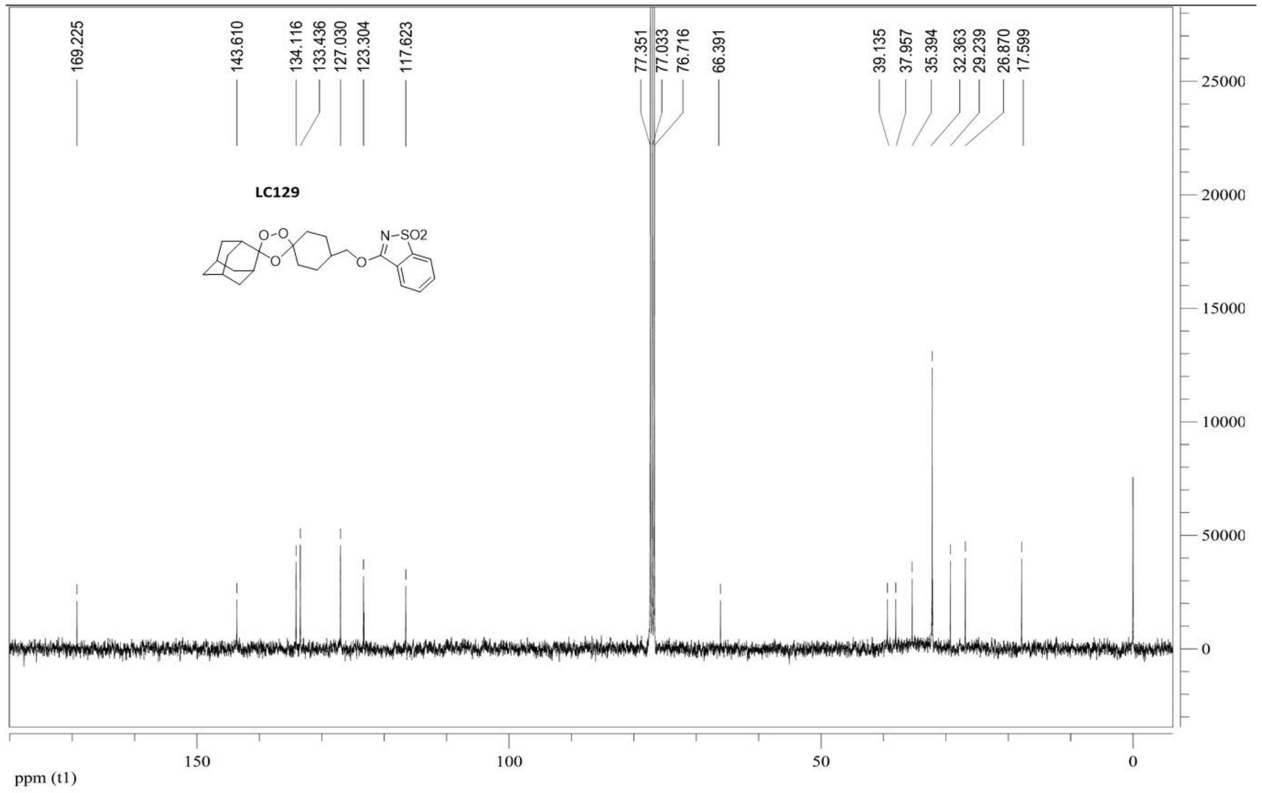

^{1}H NMR (400 MHz, $CDCl_3$) spectrum of compound **LC136**

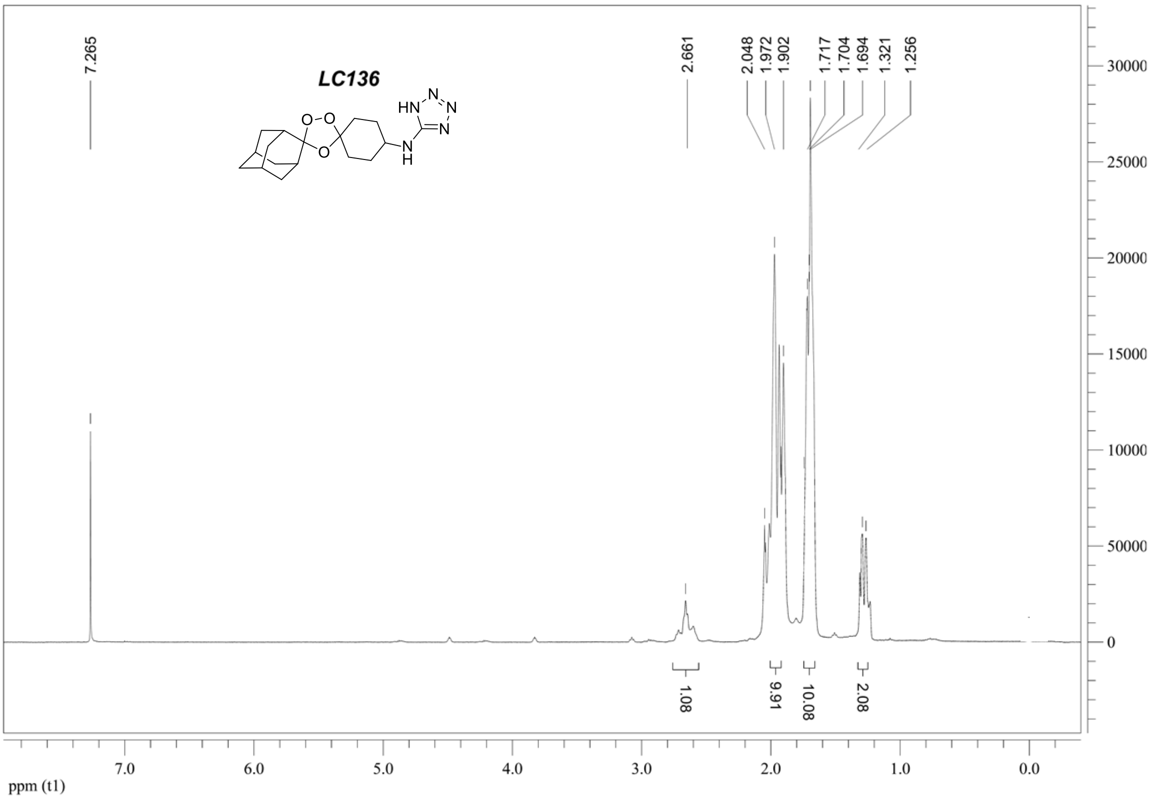

Figure 2. $^{13}C\ \{^{1}H\}$ NMR (100 MHz, $CDCl_3$) spectrum of compound **LC136**

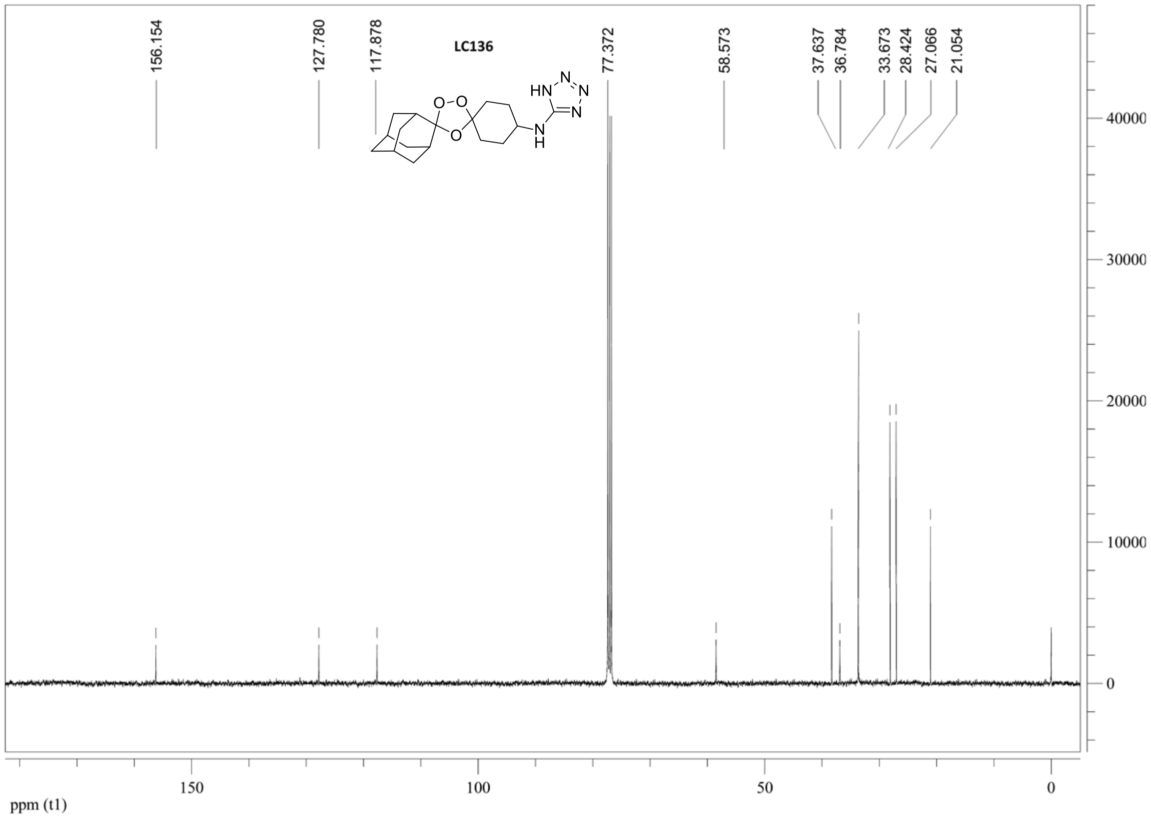

^{1}H NMR (500 MHz, $CDCl_3$) spectrum of compound **LC137**

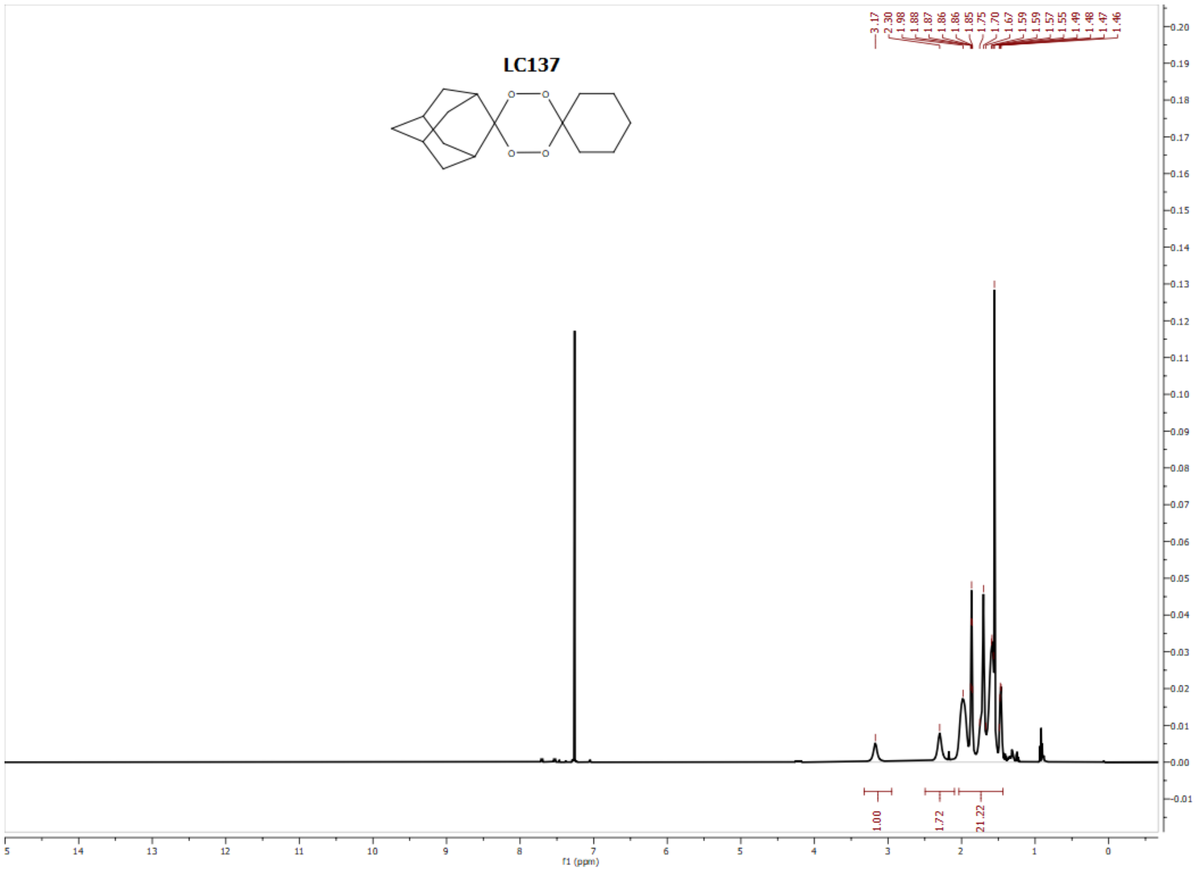

^{13}C {^{1}H} NMR (126 MHz, $CDCl_3$) spectrum of compound **LC137**

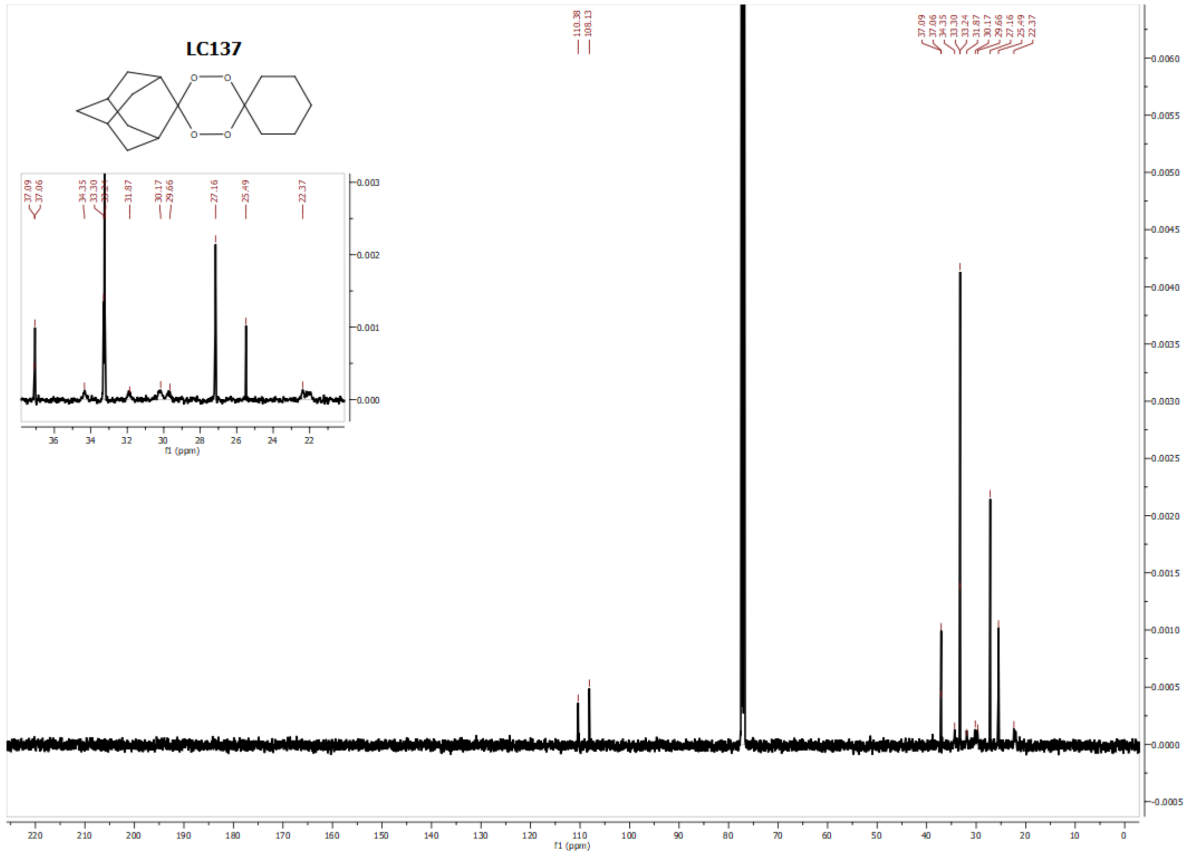

^{1}H NMR (500 MHz, $CDCl_3$) spectrum of compound **LC138**

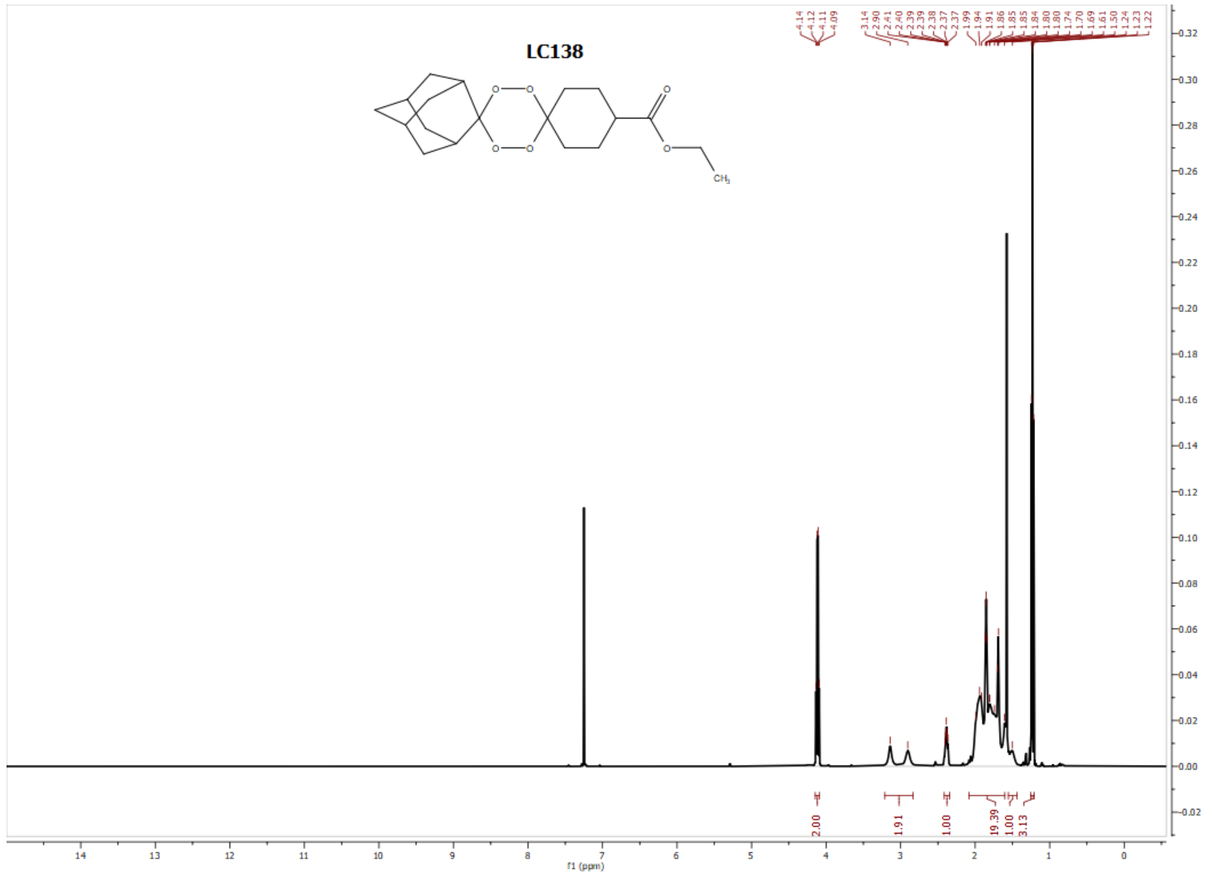

^{13}C {^{1}H} NMR (126 MHz, $CDCl_3$) spectrum of compound **LC138**

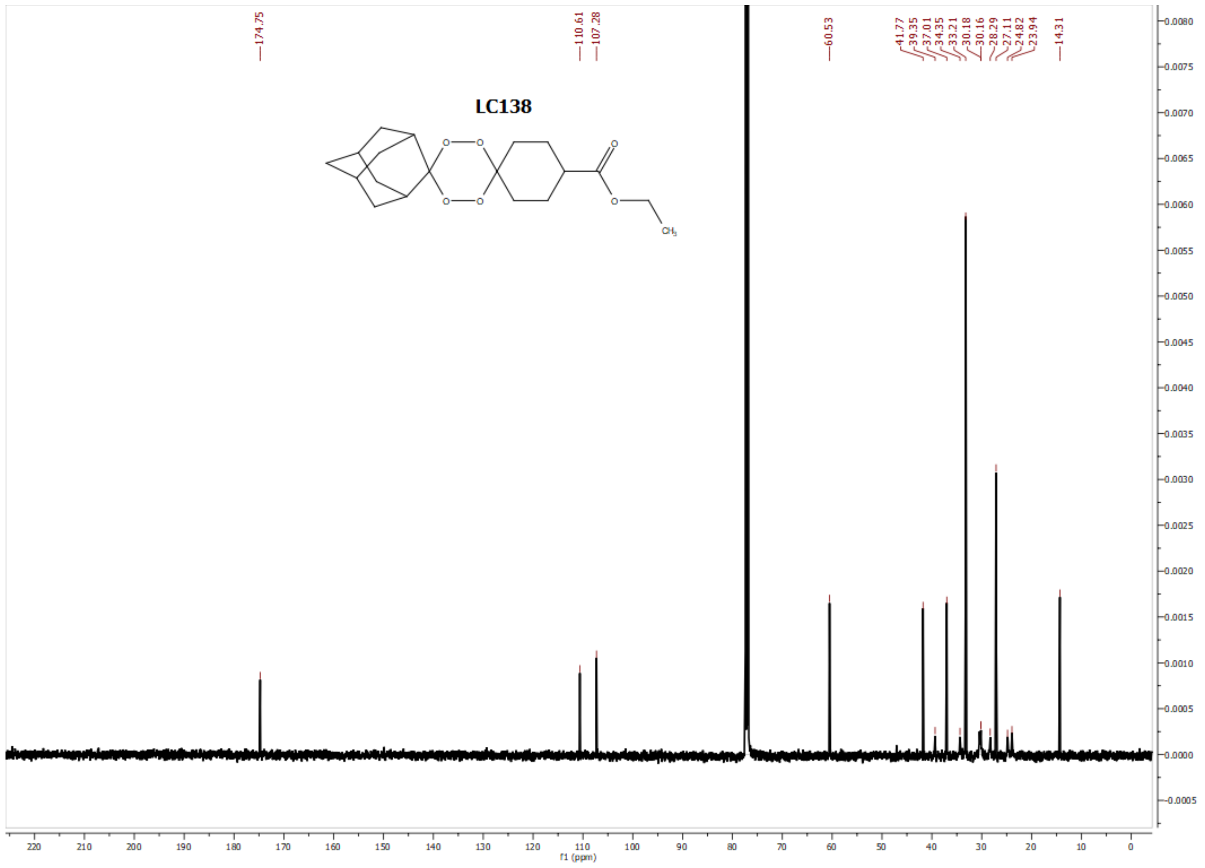

^{1}H NMR (400 MHz, $CDCl_3$) spectrum of compound **LC139**

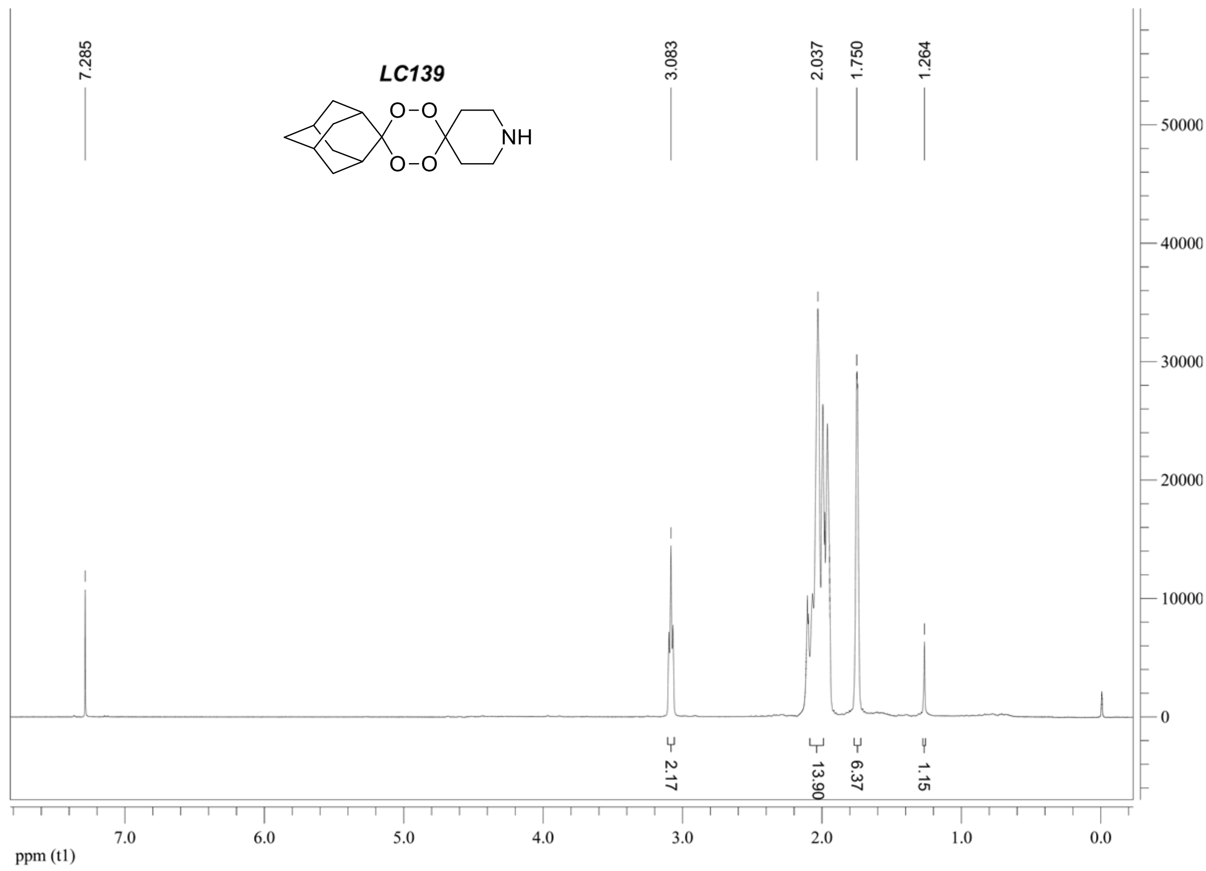

^{13}C {^{1}H} NMR (100 MHz, $CDCl_3$) spectrum of compound **LC139**

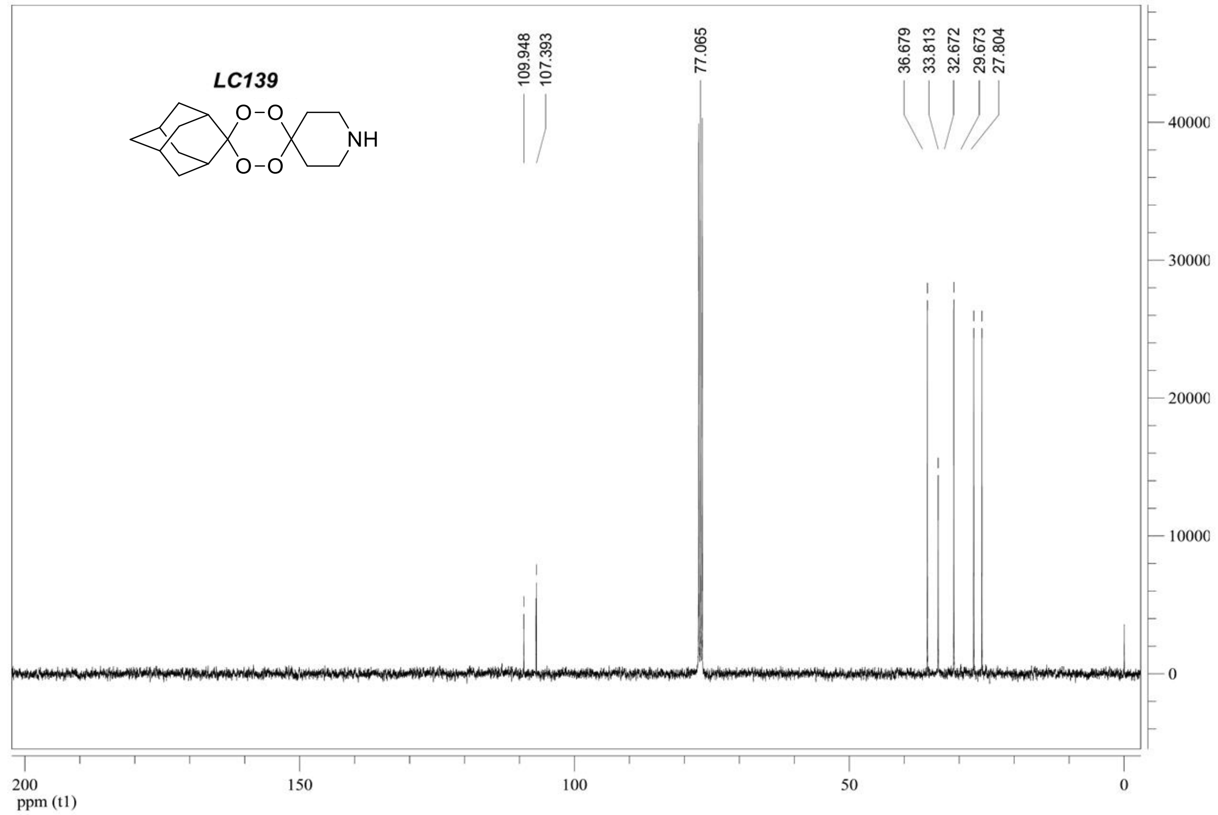

^{1}H NMR (500 MHz, $CDCl_3$) spectrum of compound **LC140**

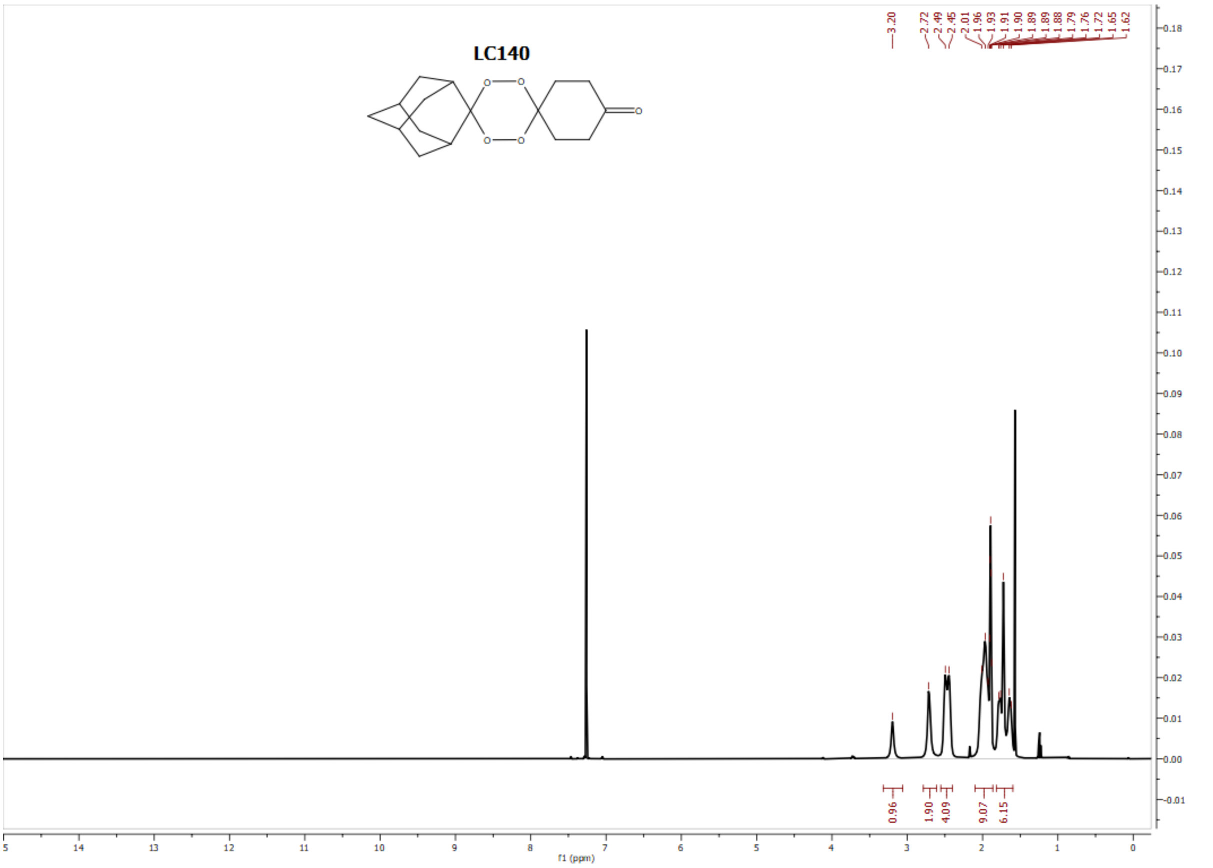

^{13}C {^{1}H} NMR (126 MHz, $CDCl_3$) spectrum of compound **LC140**

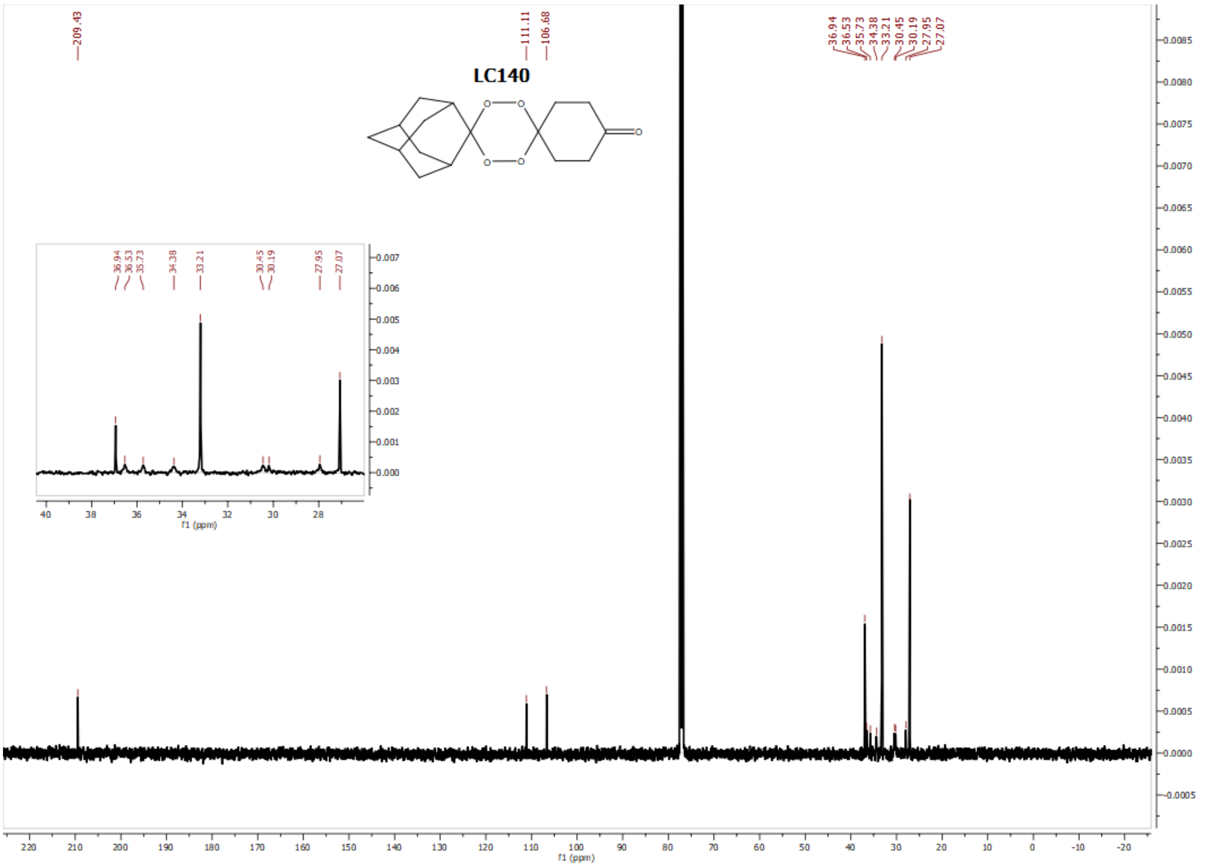

^{1}H NMR (400 MHz, $CDCl_3$) spectrum of compound **LC163**

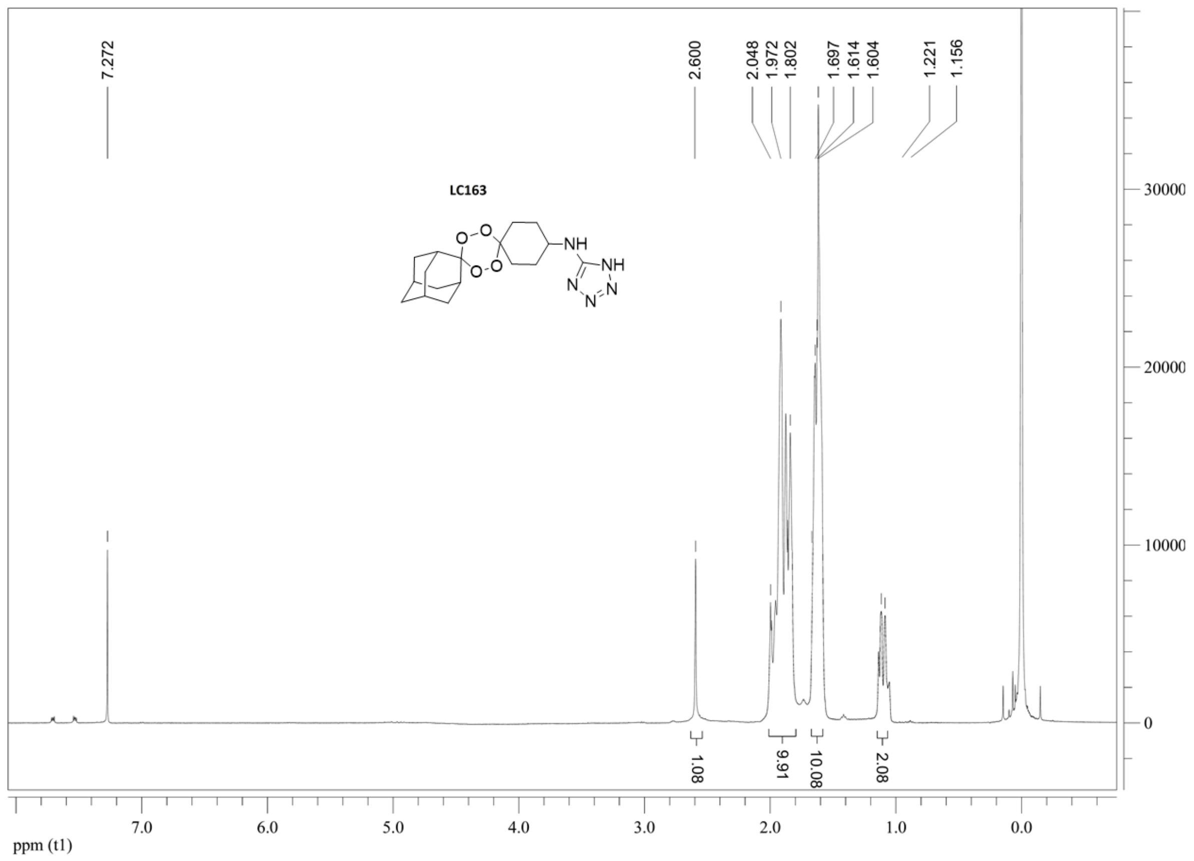

^{13}C {^{1}H} NMR (100 MHz, $CDCl_3$) spectrum of compound **LC163**

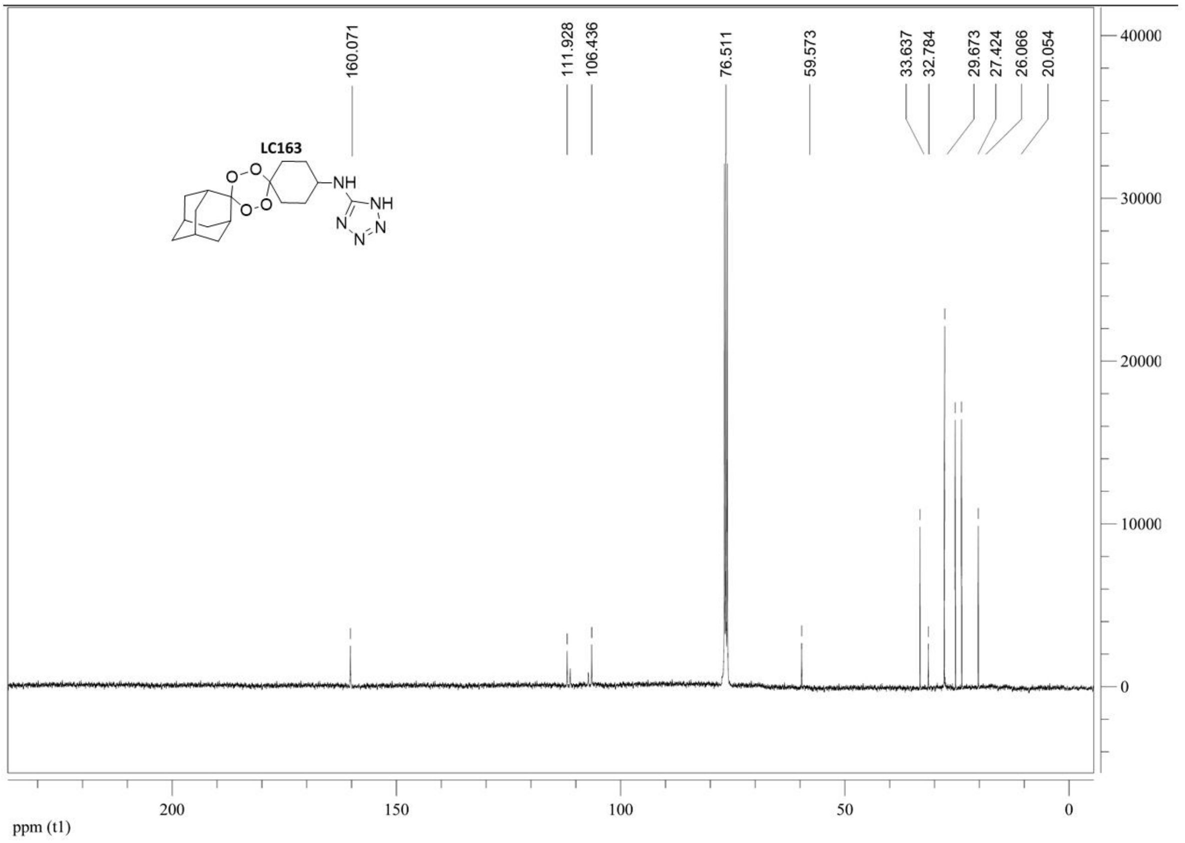

3. References

1. Ismael, A.; Henriques, M.S.C.; Marques, C.; Rodrigues, M.; Barreira, L.; Paixão, J.A.; Fausto, R.; Cristiano, M.L.S. Exploring Saccharinate-Tetrazoles as Selective Cu(II) Ligands: Structure, Magnetic Properties and Cytotoxicity of Copper(II) Complexes Based on 5-(3-Aminosaccharyl)-Tetrazoles. *RSC Adv.* **2016**, *6*, 71628–71637. https://doi.org/10.1039/c6ra15051a.
2. Kwiatkowski, M.R.; Alexanian, E.J. Nickel-Catalyzed Mizoroki–Heck-Type Reactions of Unactivated Alkyl Bromides. *Angew. Chem.* **2018**, *130*, 17099–17102. https://doi.org/10.1002/ange.201810757.
3. Lobo, L.; Cabral, L.I.L.; Sena, M.I.; Guerreiro, B.; Rodrigues, A.S.; de Andrade-Neto, V.F.; Cristiano, M.L.S.; Nogueira, F. New Endoperoxides Highly Active in Vivo and in Vitro against Artemisinin-Resistant Plasmodium falciparum. *Malar. J.* **2018**, *17*, 1–11. https://doi.org/10.1186/s12936-018-2281-x.
4. Amado, P.S.M.; Frija, L.M.T.; Coelho, J.A.S.; O'Neill, P.M.; Cristiano, M.L.S. Synthesis of Non-Symmetrical Dispiro-1,2,4,5-Tetraoxanes and Dispiro-1,2,4-Trioxanes Catalyzed by Silica Sulfuric Acid. *J. Org. Chem.* **2021**, *86*, 10608–10620. https://doi.org/10.1021/acs.joc.1c01258.
